# Structural study of the Fox-1 RRM protein hydration reveals a role for key water molecules in RRM-RNA recognition

**DOI:** 10.1093/nar/gkx418

**Published:** 2017-05-13

**Authors:** Miroslav Krepl, Markus Blatter, Antoine Cléry, Fred F. Damberger, Frédéric H.T. Allain, Jiri Sponer

**Affiliations:** 1 Institute of Biophysics, Academy of Sciences of the Czech Republic, Kralovopolska 135, 612 65 Brno, Czech Republic; 2 Regional Centre of Advanced Technologies and Materials, Department of Physical Chemistry, Faculty of Science, Palacky University Olomouc, 17. listopadu 12, 771 46 Olomouc, Czech Republic; 3 Institute of Molecular Biology and Biophysics, Department of Biology, ETH Zurich, CH-8093 Zurich, Switzerland; 4 Present address: Global Discovery Chemistry, Novartis Institute for BioMedical Research, Basel CH-4002, Switzerland

## Abstract

The Fox-1 RNA recognition motif (RRM) domain is an important member of the RRM protein family. We report a 1.8 Å X-ray structure of the free Fox-1 containing six distinct monomers. We use this and the nuclear magnetic resonance (NMR) structure of the Fox-1 protein/RNA complex for molecular dynamics (MD) analyses of the structured hydration. The individual monomers of the X-ray structure show diverse hydration patterns, however, MD excellently reproduces the most occupied hydration sites. Simulations of the protein/RNA complex show hydration consistent with the isolated protein complemented by hydration sites specific to the protein/RNA interface. MD predicts intricate hydration sites with water-binding times extending up to hundreds of nanoseconds. We characterize two of them using NMR spectroscopy, RNA binding with switchSENSE and free-energy calculations of mutant proteins. Both hydration sites are experimentally confirmed and their abolishment reduces the binding free-energy. A quantitative agreement between theory and experiment is achieved for the S155A substitution but not for the S122A mutant. The S155 hydration site is evolutionarily conserved within the RRM domains. In conclusion, MD is an effective tool for predicting and interpreting the hydration patterns of protein/RNA complexes. Hydration is not easily detectable in NMR experiments but can affect stability of protein/RNA complexes.

## INTRODUCTION

The Fox-1 family is a group of proteins that regulate alternative splicing (1). Their exact effects are tissue-specific and their proper function is essential for a developing cell (2). The Fox-1 gene was first identified in the *Caenorhabditis elegans* as a sex-determining element (3) and numerous homologs have subsequently been observed in many different organisms (2), including humans (4). At the molecular level, the Fox-1 protein functions by recognizing a 5΄-UGCAUG-3΄ sequence of the target mRNA through a single RNA recognition motif (RRM) domain which is present and highly conserved in all the tissue-specific variants of the Fox-1 protein (4,5).

The RRM is the most common RNA binding motif observed in proteins. It typically assumes a β_1_α_1_β_2_β_3_α_2_β_4_ topology with the two α-helices packed against the four-stranded antiparallel β-sheet surface (6). The exposed surface of the β-sheet is the most common (‘canonical’) RRM binding site for RNA. However, the RRM is extremely versatile and RNA binding patterns utilizing other parts of the domain (such as the α-helices, the protein loops or the unstructured chain termini) have been observed (7–10). In the case of the Fox-1 RRM, the RNA binding mode is mixed. Specifically, the UG-3’ is bound canonically by the β-sheet while the 5’-UGCA interacts with residues of the β1/α1 protein loop (4). In particular, the 5’-UGC bases wrap around a single phenylalanine side-chain. These two binding sites function independently (4).

Water is the most abundant molecule inside living cells. The structure and function of proteins and nucleic acids are dependent on hydration from their synthesis to the point of their degradation (11,12). Water molecules influence their folding and intermolecular interactions as well as their basic physical and structural properties (13,14). The ubiquity of water is structurally significant as water molecules can potentially act as both donor and acceptor of up to four hydrogen bonds. This allows individual water molecules to form ‘bridges’ between different atomic groups of the biomolecule. In the case of atomic groups belonging to two non-covalently bound molecules (e.g. in protein/RNA complexes), such water-facilitated interactions directly influence the binding affinity of the complex (15,16). The water molecules can also screen unfavorable repulsive interactions (17) and provide an entropic gain for the complex formation (18). For protein/RNA complexes, it has also been shown that those interface amino acids that interact with water are more highly conserved during evolution than other interface amino acids that do not (19).

Molecular dynamics (MD) simulation is a method for studying the structural dynamics of biomolecular complexes using simple but carefully calibrated atomistic molecular mechanics models, i.e. the force fields (20–24). The main advantage of MD simulations is the ability to observe the movement of atoms with potentially infinite spatial and temporal resolution. This makes the method particularly suitable to studying the water solvent as it does not suffer from the main limitation of experimental techniques—namely, the problem of determining the position of small, numerous and mobile water molecules in the structural experiments. In MD simulations the instantaneous position of every individual solvent molecule is always exactly known. Thus the atomistic MD simulations can be used to predict highly occupied ion sites and long-residency water bridges which have been shown to contribute to the structural dynamics of many nucleic acid systems (25–27), including the RRM protein/RNA complexes (28). On the other hand, the simulations are limited by their length (their timescale; currently 1 μs ∼ 1 ms) and the quality of the available force fields, including those used for the water (29). There are several water models commonly used in simulations of protein and nucleic acid systems, e.g. the TIPnP, SPC and OPC models (30–32). The evaluation of their performance in simulations is an important aspect of force-field development (29,33). Nevertheless, the monitoring of hydration patterns is widely considered as one of the most realistic goals of atomistic MD (20,25).

In the present study, we investigate the hydration patterns of the Fox-1 protein and its complex with RNA by using X-ray crystallography, nuclear magnetic resonance (NMR) spectroscopy and the explicit solvent MD simulations. X-ray crystallography is the leading tool for structure determination, allowing us to obtain highly accurate structures with an atomistic level of resolution, including the positions of some of the water molecules (34). NMR spectroscopy is the second most common method for structure determination of small biomolecules. It can be used to determine the biomolecular structure directly in solution, thus bypassing all those issues associated with biomolecular crystallization necessary for the X-ray experiments. However, NMR is limited by the size of the biomolecule that can be investigated, and it usually does not allow us to obtain atomic coordinates of the solvent molecules (35). The main limitation of X-ray crystallography is that it reveals only a static, ensemble-averaged picture of the hydration. However, in reality, the hydration of biomolecules is highly dynamical, with typical water residency times occurring on a sub-nanosecond time-scale (36–38). Besides the common hydration sites, folded biomolecules and biomolecular complexes can be accompanied by exceptionally variable long-residency hydration sites, which can be of structural as well as functional significance (20,21,26,39–43). In the present study, we combine the two structural experimental methods (X-ray and NMR) with the MD simulations to provide new insights into the hydration patterns of the Fox-1 RRM and RRMs in general. Specifically, we determine the X-ray structure of the free Fox-1 RRM at high resolution. We use this structure, and an earlier NMR structure of the Fox-1 RRM/RNA complex (4), as a basis for MD simulations in explicit water solvent. The resulting MD simulations predict several long-residency hydration sites in both systems. Finally, new NMR and binding experiments are used to experimentally test some of the most interesting findings revealed by the simulations.

## MATERIALS AND METHODS

### X-ray structure determination

Crystallization of the free Fox-1 RRM protein was carried out by the hanging drop vapor diffusion method at 20°C equilibrating a 5 μl drop of a solution of 3 mM protein in 20 mM NaCl and 20 mM NaH_2_PO_4_ at pH 6.5 against a reservoir containing 12.5% w/v PEG 3350, 12.5% w/v PEG 1000, 6.6% NPS, 12.5% w/v MPD and 100 mM MES at pH 6.5. Crystals were directly flash-frozen in liquid nitrogen without further cryo-stabilization. X-ray diffraction data were collected at the X06SA SLS beam line on a Pilatus 6M detector at the Paul Scherrer Institut, Villigen, Switzerland. Diffraction images were processed using XDS (44). Phases were obtained by molecular replacement using MR-Rosetta (45). Model refinement was carried out with Phenix (46). After initial refinement, waters were modeled using Coot (47). The water molecules were first filled into the 2FO-FC map with a distance to protein of between 2.0 Å and 4.0 Å. After another refinement using Phenix, waters with B-factor < 50 Å^2^ or map RMSD level < 1 e/Å^3^ were deleted. During further refinements water molecules and all other ligands were manually inspected. The statistical parameters of the data collection and refinement process are summarized in Table 1. Additional statistical parameters and visualizations of the electron density maps of selected water molecules are presented in Supplementary Table S1 and Figure S1.

**Table 1.** The high resolution X-ray structure of Fox-1 RRM

	Fox-1 RRM
**Data collections**	
Beam line	Pilatus 6M X06SA at SLS
Wavelength (Å)	0.999987
Detector distance (mm)	300
Space group	C121
Unit cell parameters (Å, °)	*a* = 68.98, *b* = 77.62, *c* = 106.39, *α* = *γ* = 90, *β* = 93.99
No. of molecules per asymmetric unit	6
No. of measured reflections^a^	18 4971 (26 029)
No. of unique reflections^a^	50 871 (7510)
Redundancy^a^	3.6 (3.5)
Completeness^a^ (%)	98.0 (97.2)
Resolution range^a^ (Å)	45.41–1.80 (1.90–1.80)
*R*_merge_^a,b^ (%)	6.1 (40.9)
*R*_meas_^a,c^ (%)	7.2 (48.3)
I/σ(I)^a^	13.4 (4.0)
**Refinement**	
Resolution range (Å)	45.41–1.80
*R*_work_^d^/*R*_free_^e^ (%)	19.4/22.7
No. of residues	
Fox-1 RRM	484
Sulfate	7
PEG	1
MES	4
Water	331
RMS(bonds) (Å)	0.008
RMS(angles) (°)	1.112
Ramachandran Most favored^f^ (%)	98.7
Ramachandran Allowed^f^ (%)	1.3
Ramachandran Outliers^f^ (%)	0
Average B-factor (Å^2^)	
Fox-1 RRM	25.4
solvent	33.2
**PDB code**	4zka

^a^Values in parentheses are for the highest resolution shell.

^b^ ${{{R}}_{{\rm{merge}}}} = \Sigma |{\rm{I}} - \langle {\rm{I}} \rangle |\Sigma \langle {\rm{I}} \rangle$

^c^ ${{{R}}_{{\rm{meas}}}} = \frac{{\sum\nolimits_h {\sqrt {m/( {m - 1} )\ } } \sum\nolimits_j {{\rm{\ }}| {{{\langle I \rangle }_h} - {I_{h,j}}} |\ } }}{{\sum\nolimits_h {\sum\nolimits_j {{I_{h,j}}} } }}$, where <I>_h_ is the mean intensity of symmetry-equivalent reflections and m is multiplicity.

^d^ ${{{R}}_{{\rm{work}}}} = \frac{{\sum {| {|{F_0}| - |{F_C}|} |} }}{{\sum {|{F_0}|} }}$, where F_0_ and F_c_ are observed and calculated structure factor amplitudes.

^e^ *R*_free_ calculated for 5% of randomly chosen reflections that were excluded from the refinement.

^f^Ramachandran Plot, as defined by the program *PROCHECK* (67).

### Molecular dynamics simulations

We have used the first conformer of the NMR ensemble (PDB ID: 2err) (4) and the chain D of the X-ray structure (PDB ID: 4zka) as the starting structures for the MD simulations of the complex and the free protein, respectively. All experimentally determined atoms were utilized. Throughout this text we utilize Fox-1 protein residue numbering as defined in Ref. (4) and used in the NMR structure of the complex (PDB ID: 2err). Note that the residue numbering differs in the deposited X-ray structure of the free Fox-1 protein (PDB ID: 4zka).

The protein molecule was described using the ff99SB, ff12SB or the ff14SB force fields (48–51). The RNA substrate was described using the ff99bsc0χ_OL3_ force field (48,49,52,53). Before each simulation, the solute was surrounded in an octahedral box of SPC/E waters (31) and neutralized with KCl or NaCl molecules (54), resulting in 150 mM excess salt concentration. These solvent conditions have been shown to be suitable for MD simulations of protein/RNA systems (24,55,56). Due to force field approximations, we did not aim to reproduce the exact solvent conditions used in the structural experiments (see Supplementary Data of Ref. (56) for discussion of differences between experimental and simulation solvent conditions). For the initial equilibration and production simulations, we have followed a standard simulation protocol for protein/RNA complexes (55). In addition, the initial stages of the complex simulations were stabilized by utilizing the experimental NMR restraints during the first 120 ns of the simulations. The specifics of this procedure are extensively discussed elsewhere (56). All simulations were conducted using the Amber 14 program suite (57).

The analyses were performed with the cpptraj program (58). VMD and PyMol were used for visualization while Raster3D was used to produce the molecular images (59,60). For the hydration analyses, we first examined the hydration pattern of the individual protein and RNA atoms by identifying the H-bonds between these atoms and the water molecules. The H-bond was considered to be present when the donor/acceptor heavy atom distance was below 3.5 Å and the donor/hydrogen/acceptor angle was >120°. This analysis indicates which atom groups participate in solute/water interactions and how often. It also allows us to identify long-term hydration sites, i.e. atom groups that interact with a single water molecule for an amount of time greater than usual (typical water exchange times in MD simulations of nucleic acids are ∼50–500 ps) (25–27,61). Finally, we identified indirect interactions between the individual solute atoms that were facilitated via water molecules (water bridges). We further validated these results with a second type of analysis in which we created density maps of the water molecules along the trajectories. In this analysis, the space around the solute was divided into small cubes (0.125 Å^3^) and the presence of a water oxygen atom inside each cube was ascertained at every trajectory frame. The resulting density map indicated regions of space where the water molecules were present most often during the simulation, i.e. with densities exceeding the bulk solvent behavior. Such results can be considered as being analogous to the electron density maps obtained from X-ray crystallography experiments and suffer from similar shortcomings, such as the averaging effects. Finally, an extensive visual analysis of the hydration sites was conducted using VMD. Note that the analysis of hydration sites in the MD simulations is a procedure that is not executed automatically like the RMSD calculations, Principle Component Analysis, cross-correlation diagrams and many others. For example, we can easily obtain a list of solute atoms forming H-bonds with the water molecules but since the entire solute is permanently surrounded by thousands of water molecules, such a simple analysis would be rather uninformative. To make a reliable interpretation, an extensive visual analysis of the MD trajectory with explicit water molecules is absolutely necessary.

Secondary structure of the protein was monitored by visual analysis (62) and by DSSP graphs (63).

### Thermodynamics integration

The thermodynamics integration (TI) method has been used to assess a free-energy impact of residue mutations on RNA binding. For the alchemical transformation, we have used the classic three-step method (57) in which we first remove the partial charges and then use the soft-core potentials (64) to handle the alchemical transformation and finally restoring the partial charges at the end. The specific protocol used in our study is described in detail elsewhere (56).

### NMR spectroscopy

Directed mutagenesis was performed on the ORF of Fox-1 RRM (amino acids 109–208) cloned in the pET28a expression vector to obtain the S122A and S155A mutated versions of the protein, respectively. The proteins were overexpressed at 37°C in *Escherichia coli* BL21 (DE3) codon plus cells in a minimal M9 medium containing 1 g/l ^15^NH_4_Cl and 4 g/l glucose, purified by two successive nickel affinity chromatography (QIAGEN) steps using an N-terminal 6× Histidine tag, dialysed against NMR buffer (10 mM phosphate buffer pH 6.5, 20 mM NaCl) and concentrated to 0.3 mM with a 10-kDa molecular mass cut-off Centricon device (Vivascience). The RNA oligonucleotide was purchased from Dharmacon, deprotected according to the manufacturer's instructions, lyophilized and re-suspended in NMR buffer. The NMR titrations were all performed in the NMR buffer at 40°C.

### SwitchSENSE experiments

Two hybridized complementary ssDNAs containing the T7 promotor followed by a GAG motif, which facilitates transcription initiation, the sequence to be transcribed and a BsaI restriction site, were cloned into the pUC19 vector. Linearization of the plasmid by the BsaI restriction enzyme was used to stop transcription at the end of the sequence of interest. RNA was then transcribed *in vitro* using T7 RNA polymerase, and purified by HPLC as described in Dominguez *et al.* (65). Thus, we obtained a long RNA sequence for which the 3’-extremity is fully complementary to the 48-nts-long ssDNAs attached to the chip, and the 5’-part corresponds to a flanking non-hybridized sequence containing a linker of 4 nts and the 5’-UGCAUGU-3’ sequence bound by Fox-1 RRM. All switchSENSE (66) experiments were performed on a DRX analyzer using MPC-48–2-Y1-S chips (both supplied by Dynamic Biosensors GmbH, Martinsried, Germany). Fox-1 RRM WT, S122A and S155A were dialysed against the T140 buffer (10 mM Tris–HCl pH 7.4, 140 mM NaCl and 0.05% tween20), which is optimized for switchSENSE measurements. In the association measurement, 250 μl of Fox-1 RRM (concentrations of 26, 39, 59, 89, 133 and 200 nM) were injected with a flow rate of 50 μl/min for a 5 min association phase. Dissociation was measured for 49 min by rinsing 2450 μl with a flow rate of 50 μl/min over the chip. All measurements were performed at 25°C. Analysis was performed with the switchANALYSIS software from Dynamic Biosensors. Normalized Dynamic Response values were obtained by subtracting the signal measured upon injection of a volume of buffer corresponding to injections performed in each titration measurement. The association and dissociation rate constants (*k*_on_ and *k*_off_) of the Fox-1 RRM interaction with the flanking 5’-UGCAUGU-3’ ssRNA sequence were derived from a global single exponential fit model by following the variation of switching speed (dynamic response) of the DNA–RNA duplex upon protein association and dissociation.

## RESULTS

### The X-ray structure of the Fox-1 protein

**Figure 1.**
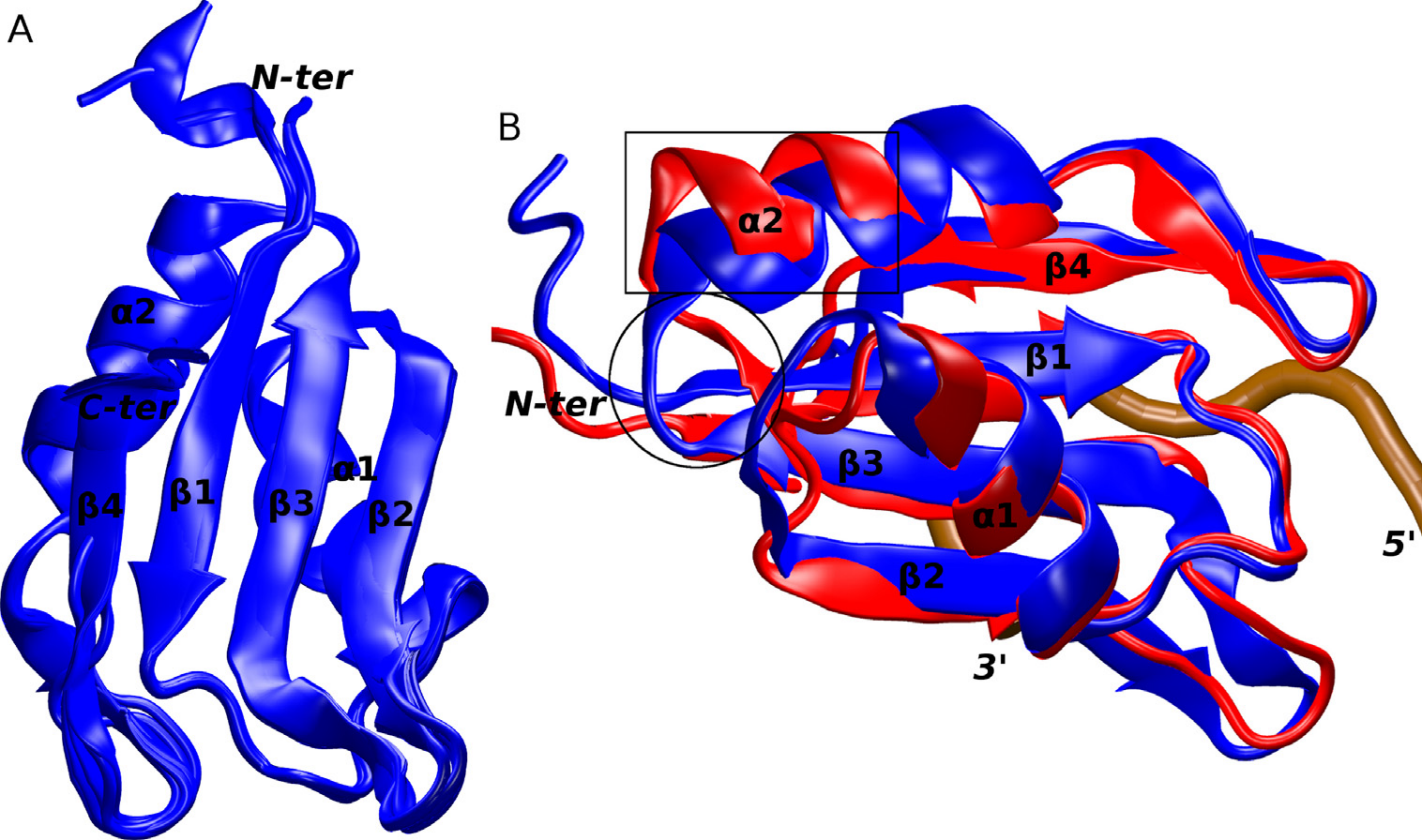
(**A**) An overlay of the six protein molecules in the X-ray structure of the free Fox-1 RRM. (**B**) An overlay of the Fox-1 RRM in X-ray (blue) and NMR (red) structures used in the MD simulations. The two differing segments are indicated by a black rectangle (α2 helix) and a circle (β3 sheet). The secondary structure of the proteins and the chain termini are labeled. The position of the RNA backbone in a formed complex is traced in brown.

The structure of the Fox-1 RRM was determined using X-ray crystallography. The protein construct used embeds residues 109–208 and the structure was solved with reflections up to 1.8 Å resolution (Table 1). There are six independent protein molecules within the crystal's asymmetrical unit that display a high degree of similarity with average backbone RMSD of ∼0.23 Å. The only structural differences (albeit minor ones) are in the flexible chain termini (Figure 1A). Also, the level of termini disorder, and therefore the number of missing residues at the chain ends, differ. In all other aspects, the individual protein molecules can be considered entirely equivalent and the following analyses apply to all the six molecules. A total of 331 unique water molecules can be found in the asymmetric unit. Counting all crystallographic contacts, the number of first solvation shell waters (closer than 3.5 Å) per individual protein molecule ranges between 63 and 80.

The X-ray structure of the free Fox-1 RRM is very similar to the NMR structure of the Fox-1 RRM complex with its target RNA that was published earlier (4). Specifically, its average backbone and heavy atoms RMSD are ∼1.6 Å and ∼3.8 Å, respectively, representing the best-fit value between the 30 NMR ensemble structures and the six molecules in the asymmetric unit of the crystal structure. The main difference between the two experimental structures is in the conformation of the protein loop β3/α2 and the adjacent ends of the β3 sheet and α2 helix (a.a. 162–173). Essentially, the area is more ‘open’ and partially unfolded in the NMR structure (Figure 1B). Curiously, it is relatively far away from the RNA binding site and therefore, the difference cannot be straightforwardly explained as an effect of the RNA binding. It is possible that the conformation seen in the X-ray structure is influenced by the local crystal packing interactions (Supplementary Figure S2). At the level of the molecular interactions, the following differences occur in this region. First, there are two salt-bridges which differ between the two structures: K142(NZ)/E164(OE) is present in the X-ray structure while K142(NZ)/D168(OD) is found in the NMR structure. Second, there are five hydrogen-bond interactions which are present in the X-ray structure but absent in the NMR structure, namely D168(N)/N165(OD1), N165(ND2)/D168(OD), K113(O)/S166(N), Q115(O)/S166(N) and Q115(O)/S166(OG). The third difference is in the F163 side-chain which has a different conformation due to a difference in its χ_1_ dihedral angle: χ_1_ = −77° (*gauche* −) in the X-ray structure, whereas in the NMR ensemble it is in *trans* with an average χ_1_ = −168° (4) (Supplementary Figure S3). However, after a manual re-analysis of the original NOESY spectra recorded with the Fox-1 protein/RNA complex (4), we suggest that the F163 side-chain difference was due to errors in the ATNOS/CANDID automatic assignment of a small number of ambiguous intra-protein NOEs originating from F163 protons. Note that this potential error in the NMR structure should not have any effect on the MD analysis for the protein–RNA interface, as it is localized far from the RNA binding site.

### MD simulations of the free Fox-1 RRM and of the protein/RNA complex

**Figure 2.**
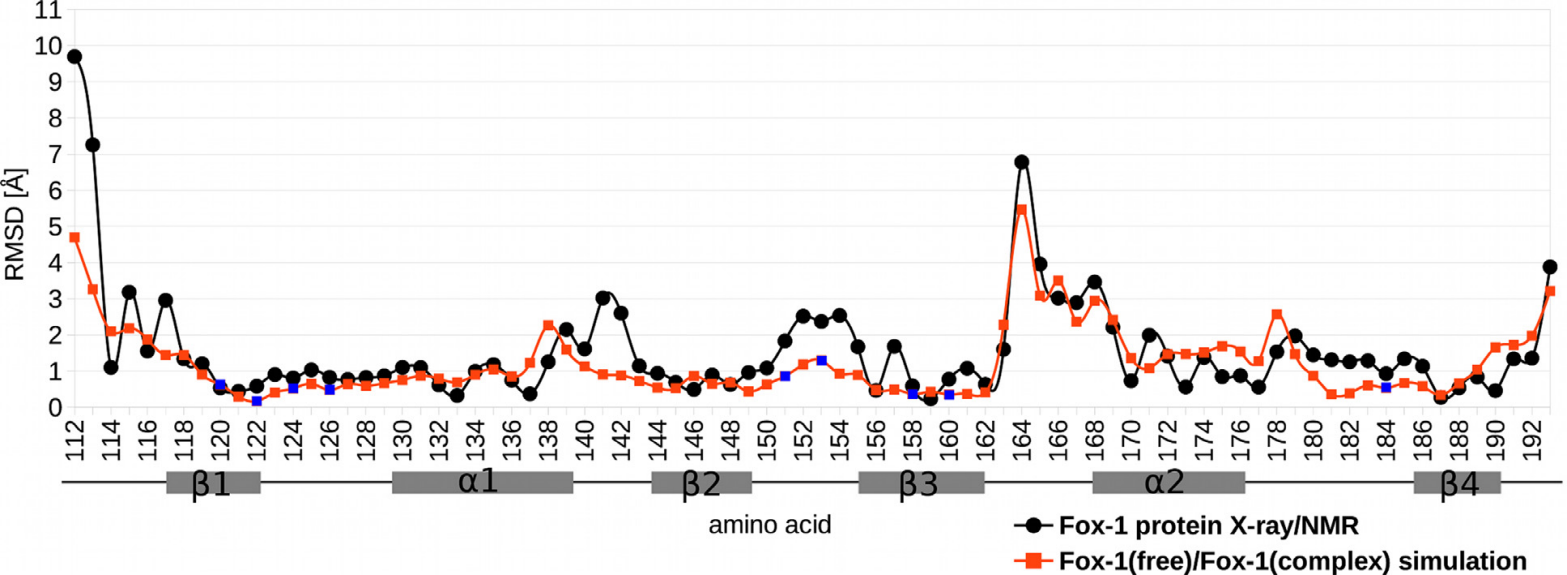
The graph of the average per residue heavy atom RMSD between the best-fit X-ray and NMR structures (black line) and between the averaged structures from the Fox-1(free)_12_1 and Fox-1(complex)_12_1 simulations (red line). The secondary structure elements of the protein are shown below the x-axis. The blue data points indicate amino acids that interact with the RNA within the protein/RNA complex.

MD simulations of the free Fox-1 RRM were conducted based on the X-ray structure of the isolated Fox-1 RRM (PDB ID: 4zka)—henceforth referred to as ‘Fox-1(free) simulations’. The simulations of the protein/RNA complex were based on the NMR structure (PDB ID: 2err) (4) and are referred to as ‘Fox-1(complex) simulations’. The total run-time of MD simulations in our work is 60.1 μs, with some individual runs extended up to 10 μs. During the course of the Fox-1(free) and Fox-1(complex) simulations, the MD trajectories displayed similar differences as observed in the two starting structures. The presence of bound RNA did not significantly alter the conformation of the interface amino acids (Figure 2). Instead, the largest simulation differences were observed in the β3/α2 loop region, which were also significantly different in their starting structures (see above). Both of these distinct conformations were fully maintained in their respective simulations. The region is far from the RNA binding site and thus we initially suspected that the differences may have been due to the different experimental methods (X-ray versus NMR) used to obtain the starting structures. To verify this assumption, we performed additional simulations of the free Fox-1 RRM based on the protein/RNA complex structure with the RNA removed by molecular modeling—henceforth referred to as ‘Fox-1(free*) simulations’. Results of these simulations are primarily described in Supplementary Data. The idea behind the Fox-1(free*) simulations was that the β3/α2 loop region conformation would either be maintained, thus indicating that it is independent of the RNA, or eventually converge to the conformation seen in the X-ray structure of the free Fox-1 RRM, thus indicating RNA dependence. It is, however, also possible that the entire β3/α2 loop region difference may have been affected by the incorrect side-chain dihedral assignment of the F163 residue in the initial structure of the protein/RNA complex, as noted above. If this is the case, the MD simulation could, in principle, correct the structure, provided the simulations are long enough. However, as discussed in the Supplementary Data, our μs-scale simulations were not capable of unifying the behavior of the β3/α2 loop region between trajectories initiated from X-ray and NMR structures. Since this region is far away from the protein–RNA interface, we made no further efforts to resolve the difference.

### Important hydration sites in the Fox-1 RRM

We conducted a hydration analysis of the Fox-1 system (see ‘Materials and Methods’ section) utilizing the experimental X-ray structure of the free protein, the MD simulations of the free protein (‘Fox-1(free) simulations’) and the MD simulations of the protein/RNA complex (‘Fox-1(complex) simulations’). We analyzed the differences in hydration among these three data sources. The simulations based on the protein/RNA complex with the removed RNA (‘Fox-1(free*) simulations’) were merely used to clarify whether the observed hydration differences were due to the presence of the RNA molecule or due to other unrelated structural differences between the X-ray and NMR structures (see above and Figures 1 and 2). The following results are mainly based on the simulations using the ff12SB protein force field which showed the best performance (see below) and which were then extended to a multiple microsecond time-scale (Fox-1(complex)_12_1 and Fox-1(free)_12_1; see Table 2). All the other simulations (including the simulation with NaCl used instead of KCl; see Table 2) were, nevertheless, also monitored in order to ensure that, qualitatively, the same behavior was achieved.

**Table 2.** List of simulations

simulation name^a,b^	length[ns]
Fox-1(complex)_12_1	10 000
Fox-1(complex)_12_2	1000
Fox-1(complex)_12_3	1000
Fox-1(complex)_12_4	1000
Fox-1(free)_99_1	1000
Fox-1(free)_99_2	500
Fox-1(free)_12_1	8000
Fox-1(free)_12_2	500
Fox-1(free)_14_1	500
Fox-1(free)_12_frozen^c^	300
Fox-1(free)_12_NaCl^d^	1000
Fox-1(free*)_99_1^b^	1000
Fox-1(free*)_99_2^b^	500
Fox-1(free*)_12_1^b^	7000
Fox-1(free*)_12_2^b^	500
Fox-1(free*)_14_1^b^	500
Fox-1(free*)_12_frozen^b,c^	300
Fox-1(complex)_12_S155A^e^	1000
Fox-1(complex)_14_S155A^e^	1000
Fox-1(complex)_12_S155A_TI^e,f^	54 × 200
Fox-1(complex)_12_S122A^e^	500
Fox-1(complex)_12_S122A_2^e^	1000
Fox-1(complex)_14_S122A^e^	1000
Fox-1(complex)_12_S122A_TI^e,f^	54 × 200

^a^The numerals ‘14’, ‘12’ and ‘99’ in the simulation name indicate ff14SB, ff12SB and ff99SB protein force-field versions, respectively. For the RNA, the ff99bsc0χ_OL3_ force field was used in all simulations.

^b^The “Fox-1(free*)” simulations were based on the Fox-1 protein/RNA complex structure (PDB: 2err) with the RNA removed.

^c^The solute was restrained in its initial conformation by positional restraints.

^d^NaCl was used in the simulation instead of KCl.

^e^The S155A and S122A mutations, respectively, were introduced into the system by molecular modeling. The structure from the 1000 ns time point of the Fox-1(complex)_12_1 simulation was used as the starting structure.

^f^The TI (thermodynamics integration) calculations consisted of 54 independent simulations, each lasting 200 ns.

The water molecules were initially positioned in all systems by surrounding the solute atoms in a truncated octahedral-shaped water box (see the ‘Materials and Methods’ section). This standard procedure ensures that there are no water/solute atomic clashes; but otherwise, the water molecules are placed around the solute in a random pattern. Their correct positioning within the individual hydration sites occurs spontaneously during equilibration or in the initial phases of the MD simulations. The same procedure was applied in the Fox-1(free) simulations except that we also utilized all the water molecules identified in the X-ray experiment, adding the rest of the water molecules as the bulk solvent. In other words, some of the hydration sites were known in the experiment and were thus preformed in the initial structure of the Fox-1(free) simulations.

The water molecules in the MD simulations can be divided into three groups: (i) rapidly exchanging ‘bulk’ waters; (ii) surface waters forming the first hydration shell of the solute; and (iii) the waters forming bridges between the solute atoms (28). Our analysis focused on the last type of water molecules as they have a direct influence on the biomolecular structure (68). Moreover, a water bridge that forms between bound protein and RNA, can contribute to their binding affinity.

### Hydration sites in the Fox-1 RRM—the X-ray structure

The X-ray structure clearly reveals water molecules around the protein. However, determination of solvent molecules by biomolecular X-ray crystallography is more ambiguous than the determination of solute atoms (69–73). This is because the hydration network is affected by subtle biochemically irrelevant differences in the crystal packing and the genuine thermal fluctuations of the, often flexible, hydration shell.

To overcome this issue in our study of the Fox-1 RRM, we have utilized the fact that there are six independent protein molecules within the asymmetric unit. While these six individual protein molecules are very similar, their solvation shells are not. Thus, we have superimposed the individual protein molecules and computed a water density grid to identify the positions where the water molecules are consistently found in more than one molecule. We have also computed a list of atoms coordinating the water molecules in each molecule. Our assumption was that those hydration sites present in multiple protein molecules should be the most significant ones. We suggest this as an efficient approach in identifying significant hydration patterns in cases where there is more than one crystal structure of the same biomolecule available and/or when there are more biomolecules in the asymmetric unit. The hydration sites identified in the X-ray structure in more than one protein molecule are summarized in Table 3. Our initial assumption was subsequently supported by MD simulations as the hydration sites present in all six crystallographically independent protein molecules were also the most stably occupied in the MD simulations (see below and Supplementary Figure S4).

**Table 3.** The list of hydration sites identified in the six crystallographically independent molecules of the X-ray structure of the free Fox-1 RRM (PDB: 4zka)

#		X-ray molecules						
	interacting atoms	1	2	3	4	5	6	^#^✓^a^
1	**H120(ND1)/E187(O)/**	✓	✓	✓	✓	✓	✓	6
	**N189(ND2)/H120(O)**							
2	**S155(OG)/F128(O)/P125(O)**	✓	✓	✓	✓	✓	✓	6
3	**D168(OD)/N165(N)/K142(O)**	✓	✓	✓	✓	✓	✓	6
4	**D132(O)**	✓	✓	✓	✓	✓	✓	6
5	**R118(NH2)/E147(OE)/D145(OD)**	✓	✓	✓	✓	✓	✓	6
6	I143(N)	✓	✓	×	✓	✓	×	4
7	R173(NH1)/V188(O)	✓	✓	×	✓	×	×	3
8	R118(NE)/T162(OG1)	✓	✓	×	×	✓	×	3
9	I124(O)	✓	×	×	✓	×	✓	3
10	K156(O)	✓	×	×	✓	×	✓	3
11	R127(N)	×	✓	✓	×	×	✓	3
12	E187(OE)/N189(ND2)	✓	×	×	✓	×	×	2
13	S155(O)/N151(OD)	✓	×	×	✓	×	×	2
14	K185(O)/E187(OD)	✓	×	×	✓	×	×	2
15	D170(OD)/R173(NE)	×	✓	×	✓	×	×	2
16	R127(NH1)/D132(OD)	×	×	✓	×	×	✓	2
17	F140(O)/R171(NE)	✓	×	×	✓	×	×	2
18	D130(OD)/V146(O)	×	✓	×	×	×	×	1

^a^The ‘^#^✓’ number indicates in how many of the protein molecules within the asymmetrical unit was the water molecule present in the specified location. The ✓ and ✗ symbols indicate presence or absence of the water molecule, respectively. Only hydration sites found in at least two molecules are presented (with one exception). The water molecules that merely constitute the solvation shell of exposed charged amino acids are omitted.

### Hydration sites in the Fox-1 RRM—the MD simulations

The hydration sites observed in the MD simulations of the Fox-1 RRM can be divided between those observed in all simulations and those seen either only in the Fox-1(free) or only in the Fox-1(complex) simulations. The latter can be further divided into hydration sites sensitive to RNA binding and those that are sensitive to a different conformation of the β3/α2 loop in the X-ray and NMR structures (see above and Figures 1 and 2). However, it is possible that the conformation of the β3/α2 loop seen in NMR structure is erroneous. Therefore, the unique hydration sites of the β3/α2 loop observed in NMR structure simulations are summarized in the Supplementary Data.

Note that our MD simulations show a number of transient salt-bridges (74) between arginine or lysine and aspartate or glutamate side-chains. In simulations, these residues usually form a direct salt-bridge interaction. However, they sometimes become temporarily water-mediated with a highly coordinated system of bridging water molecules between them (Supplementary Figure S5). In general, we excluded such water bridges from our analysis unless they were related to RNA binding or were otherwise significant.

#### Hydration sites universally present in all simulations of Fox-1 RRM (free protein and protein/RNA complex)

Hydration sites are not easily detectable by NMR measurements; nevertheless, the hydration sites of the protein/RNA complex could be quickly established using the MD simulations. We found that most hydration sites in the protein/RNA complex were identical to those observed in the X-ray structure of the free Fox-1 RRM and then seen in its MD simulations. This confirms that MD simulation is a very effective tool for reliably predicting hydration sites.

**Figure 3.**
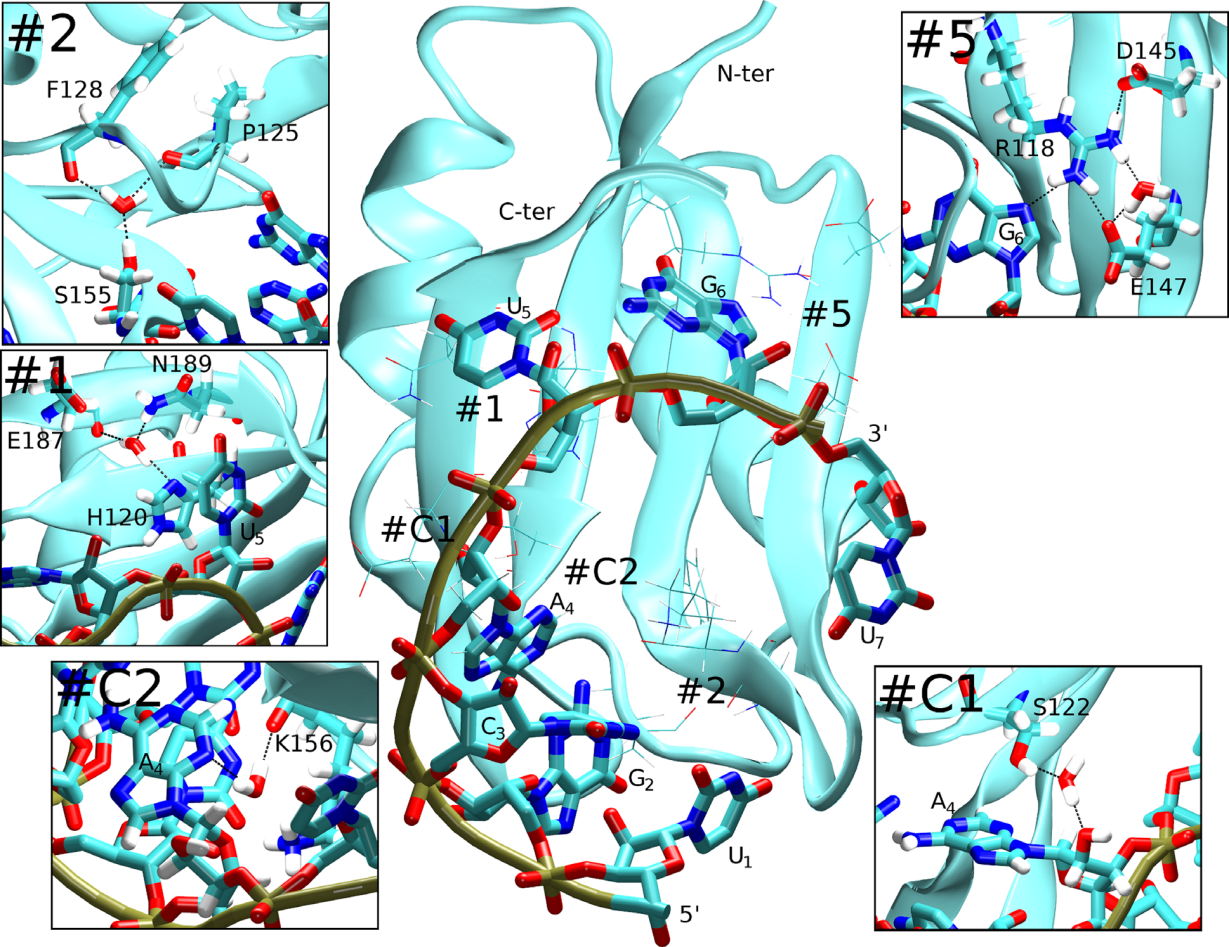
Selected hydration sites observed in the simulations of the Fox-1 RRM system. The RNA nucleotides and the chain termini are labeled. The boxes show details of the individual hydration sites with the water coordination indicated by dotted black lines and the coordinating residues labeled. Sites #1 and #2 were seen in both the Fox-1(free) and Fox-1(complex) simulations. Sites #C1 and #C2 were related to the binding of the RNA molecule and occurred exclusively in the simulations of the complex. Site #5 behaved as a transient salt-bridge in the free protein. Its behavior (and hydration pattern) was altered by the RNA binding.

Our data suggest that the most significant hydration site of the Fox-1 RRM is the water molecule #2 of Table 3 which is coordinated by S155(OG), P125(O) and F128(O) atoms (Figure 3, #2). In the X-ray structure, it is found in all six protein molecules (Table 3) and it was also observed in all MD simulations. In the Fox-1(complex) simulations, it had unusually long water-exchange times of hundreds of nanoseconds (Table 4). Common water-binding sites have binding times of 50–500 ps in comparable simulations (25,37,61). In the Fox-1(free) simulations, we observed faster exchange rates occurring in the tens of nanoseconds (Table 4), which, however, should still be considered as extremely tightly bound waters. Structurally, this hydration site stabilizes the position of the β2/β3 loop relative to the β1/α1 loop, two loops which are critical to RNA recognition (4). In the Fox-1(complex) simulations, no NOE violations were associated with the presence of this hydration site. Therefore, the simulations correctly predicted (in the Fox-1(complex) simulations) and reproduced (in the Fox-1(free) simulations) the existence of this hydration site. To further verify the significance of this hydration site for RNA binding, we prepared a Fox-1(S155A) RRM mutant protein for additional NMR experiments and MD simulations (see below).

**Table 4.** The list of important hydration sites in the Fox-1(complex)_12_1 and Fox-1(free)_12_1 simulations

Interacting atoms	Fully formed water bridge (%)^a^		Maximum water binding time (ns)		#^b^
	Free protein	Complex	Free protein	Complex	
**Sites observed in both simulations**					
S155(OG)/P125(O)/F128(O)	78	92	64	327	2
H120(ND1)/E187(O)/ H120(O)/N189(ND2)	66	54	7	19	1
D132(O)	78	72	4	3	4
R118(sc)/E147(OE)	26	44	9	5	5
R118(sc)/D145(OD)	37	0	8		5
R173(sc)/V188(O)	22	19	3	4	7
D130(OD)/V146(O)	46	40	3	2	18
**Sites observed in the Fox-1(free) simulation only**					
K142(N)/D168(OD)	44		9		3
F140(O)/D168(OD)	38		2		3
**Sites observed in the Fox-1(complex) simulation only**					
A_4_(O2΄)/S122(OG)		55		83	C1
A_4_(N3)/K156(O)		17		20	C2
C_3_(OP2)/A_4_(N7/N6)		54		3	C3
G_2_(O6)/R127(N)		38		3	C4
E164(OE)/S166(O)^c^		8		58	C5
K142(O)/F163(O)^c^		23		25	C6
D170(OD)/R173(sc)^c^		9		19	C7

^a^Fraction (in %) of the simulation time where the water bridge is fully formed. Note that on a microsecond simulation time-scale, the individual water bridges can be temporarily abolished due to reversible local solute structure fluctuations—see the main text for further explanation. The coordinating atoms can either move closer to each other, thus establishing a direct H-bond, or farther apart, allowing multiple bulk waters to come between them.

^b^Numerical designation of the specific hydration site, as used in the text and in the figures.

^c^Hydration site was related to the possibly erroneous conformation of the β3/α2 loop in the protein/RNA complex structure, see Supplementary Data.

A second salient simulation hydration site was represented by a water molecule coordinated by atoms H120(ND1), N189(ND2) and E187(O) or H120(O). It is present in all protein molecules of the X-ray structure (Table 3, #1) and was consistently found in all simulations. It had approximately a nanosecond time-scale exchange rate (Table 4) and the N189(ND2) atom was sometimes not involved in coordination depending on the random fluctuations of the N189 side-chain. This hydration site maintains the position of the H120 side-chain which is involved in the protein/RNA interaction where it stacks with the U_5_ nucleobase (4) (Figure 3, #1). Even in the Fox-1(free) simulations, the water-mediated interaction between the H120 and the other protein residues could hold the H120 side-chain in a conformation identical to that of the protein/RNA complex. This water-mediated interaction could therefore be important for RNA binding by restricting the H120 side-chain movements and increasing the chances of a conformational capture by the RNA molecule.

Yet another hydration site near atom D132(O) (Table 3, #4) is present in all six molecules of the X-ray structure and was consistently formed in all simulations. Its water molecules were merely interacting with a partially exposed carbonyl oxygen of the α1 helix, with, apparently, no specific role (Supplementary Figure S6, #4).

There was also a dynamical hydration site with the water coordinated by R118(sc) and E147(OE) or alternatively by R118(sc) and D145(OD) atoms (Table 3, #5). The hydration site was present in the Fox-1(free) simulations as one of the common (see above) transient salt-bridges. However, the R118 interaction with the G_6_ nucleotide in the complex altered the hydration site simulation behavior. Specifically, the R118/D145 contact was always direct in the Fox-1(complex) simulations, without any water mediation. The R118/E147 contact was stabilized as a direct interaction of the E147 side-chain with one of the arginine side-chain nitrogen atoms while there was a water bridge connecting it to the second arginine nitrogen (Figure 3, #5). Significantly, in the Fox-1(free*) simulations, this hydration site behavior quickly converged to that of the Fox-1(free) simulations. In conclusion, the presence of RNA stabilized a single hydration pattern in this region whereas it was variable in the free protein.

Yet another water molecule was coordinated by atoms R173(*sc*) and V188(O) (Table 3, #7). It was seen in only three protein molecules of the X-ray structure (Table 3) but in all MD simulations. It helped to anchor the α2 helix to the β4 sheet (Supplementary Figure S6, #7). Another water molecule was coordinated by atoms D130(OD) and V146(O). It is seen in the X-ray structure (albeit only in a single model, #18) and in all simulations. It helped to anchor the α1 helix to the β2 strand (Supplementary Figure S6, #18).

#### Hydration sites observed only in the Fox-1(free) simulations

These hydration sites were observed in the Fox-1(free) simulations but due to structural differences between the X-ray and NMR starting structures (the β3/α2 region; unrelated to the RNA substrate presence, see above and Figures 1 and 2), they were not seen in the Fox-1(complex) simulations. One water molecule was coordinated by K142(N) and D168(OD) atoms with occasional involvement of the N165(N) atom. It was present in the X-ray structure in all six protein molecules (Table 3, #3) as well as in the Fox-1(free) simulations. A second hydration site facilitated contact between the D168(OD) and F140(O) atoms (Supplementary Figure S6, #3).

#### Hydration sites observed only in the Fox-1(complex) simulations

The main hydration sites related to RNA binding included a water coordinated by S122(OG) and A_4_(O2΄) atoms (Figure 3; Table 4, site #C1). This water-mediated protein/RNA interaction was dominant in simulations and alternated with a rare (population <1%) direct S122(OG)/A_4_(O2΄) H-bond. It could be potentially significant since its residency time was long (the longest water-binding event seen in our simulations was 83 ns) and A_4_ did not possess any other direct interactions with the Fox-1 protein (4). To explore this hydration site further, and its significance for RNA binding, we prepared a Fox-1(S122A) mutant protein for additional NMR experiments and simulations (see below).

Another water molecule was coordinated by atoms K156(O) and A_4_(N3), mediating a second protein/RNA interaction with the A_4_ nucleotide which also had fairly long water-binding times (Figure 3; Table 4, #C2). We detected two additional water molecules related to A_4_ coordinated by either C_3_(OP2) and A_4_(N7) or C_3_(OP2) and A_4_(N6) atoms (Table 4, #C3). This water-mediated interaction further stabilized the A_4_/G_2_ intramolecular base-pair (Supplementary Figure S6, #C3). The last detected hydration site included two water molecules located near R127(N) and G_2_(O6) atoms. This hydration site extended the specific recognition between the protein and the RNA by allowing the O6 atom of G_2_ to be additionally recognized by the R127(N) atom (Supplementary Figure S6, #C4).

Altogether, based on the simulations, several water molecules seem to play a role in the RNA recognition of Fox-1 RRM. Remarkably, there was a cluster of water molecules around the A_4_/G_2_ non-canonical intramolecular base pair that forms upon RNA binding (Supplementary Figure S7).

### Population of the hydration sites

Early MD simulation studies reporting structured hydration around folded RNAs and in protein–RNA complexes have indicated hydration site occupancies typically at 100% (see, e.g. Refs. (26,42)). In contrast, Table 4 reports substantially lower occupancies. This is because Table 4 reports occupancies of fully formed water bridges, as defined by the interacting solute atoms. When, during the solute fluctuations, there are structural changes of the network of donors and acceptors, the listed hydration sites disappear. The exposed donors and acceptors obviously still remain fully hydrated (by common short-residency waters), but with different water molecule configurations, and then they are not counted. Earlier simulations were typically conducted over too short a time-scale (1–25 ns) to reveal such larger solute fluctuations. Our longer simulations therefore represent a more realistic view of the structured hydration and allow us to evaluate binding times for the water molecules at the protein–RNA interface, including the structural sub-states.

### Experiments and simulations with mutated residues

To confirm the simulation results, we have experimentally investigated two of the predicted hydration sites, namely, near residues S155 and S122 (see above and Figure 3, #2 and #C1). The S155 site maintains the position of the β2/β3 protein loop in relation to the β1/α1 protein loop. It could affect RNA binding due to its proximity to the protein/RNA interface. It is consistently seen in the X-ray structure of the free Fox-1 RRM, in Fox-1(free) simulations as well as in all Fox-1(complex) simulations. The S122 hydration site forms a water bridge between the S122(OG) atom and the A_4_(O2΄) atom of the RNA in the Fox-1(complex) simulations. The A_4_ nucleotide does not form any direct H-bond interactions with the protein; the S122 hydration site could thus influence the protein/RNA affinity. The selection of the S155 and S122 hydration sites was also motivated by the fact that both of them utilize serine's side-chain hydroxyl group for the water coordination and the sites can thus be straightforwardly abolished by mutating the serine into alanine.

Changes in the stability of the Fox-1 protein/RNA complex (in terms of Gibbs free-energy of complex formation) as a result of various alanine single-point mutations are well documented experimentally (4). However, the S122A and S155A mutations have not been studied before. Here, we investigated these two point mutants by both experimental and simulation techniques. The S155A and S122A mutations were separately introduced into the wild-type (WT) Fox-1 protein. The affinity of the mutants for the Fox-1 target RNA sequence was then measured and compared with the WT complex. For the simulations with mutated proteins, we have used the end (1μs) of the well-behaving Fox-1(complex)_12_1 simulation as the starting point (Table 2). To verify reproducibility, multiple simulations were conducted. We analyzed the structural impact of the mutations in comparison with simulations of the native system. Furthermore, we used the TI method to estimate the difference between the RNA binding free-energy of the mutated and WT proteins. For details of the TI method, see reference (56). For details of the experimental techniques, see the ‘Materials and Methods’ section.

### Simulations of the Fox-1(S155A) system

As indicated by the WT simulations, S155 participates in a major hydration site of the Fox-1 RRM with the S155(OG1) atom coordinating a water molecule (Figure 3, #2). While the RNA nucleotides do not directly participate, there are important proximal amino acids constituting the protein/RNA interface. Namely, F126 interacts with three nucleotides (4,56) (Supplementary Figure S8). In the Fox-1(complex)_12_S155A simulation, at a time-point 765 ns into the simulation, the F126 aromatic ring flipped away from its starting position, irreversibly disrupting the associated hydrophobic pocket. In contrast, the Fox-1(complex)_14_S155A simulation showed no such event. Because the F126 flip was also observed in some of the earlier simulations of the WT Fox-1 system (56), the structural change may be due to random sampling and may be unrelated to the S155A mutation. In other words, the result cannot be considered as statistically significant. We did not have enough simulation time to fully converge the behavior but we suggest that, compared to the WT, the Fox-1 S155A complex may remain structurally unchanged, although the β2/β3 protein loop perturbations may be more frequent after the mutation. The free-energy penalty for RNA binding predicted by the TI calculation was ∼1.2 ± 0.3 kcal/mol compared to the WT. In conclusion, the simulations predict a minor decrease in RNA binding affinity of the S155A mutant with no change of the binding mode.

### Experiments on the Fox-1(S155A) system

**Figure 4.**
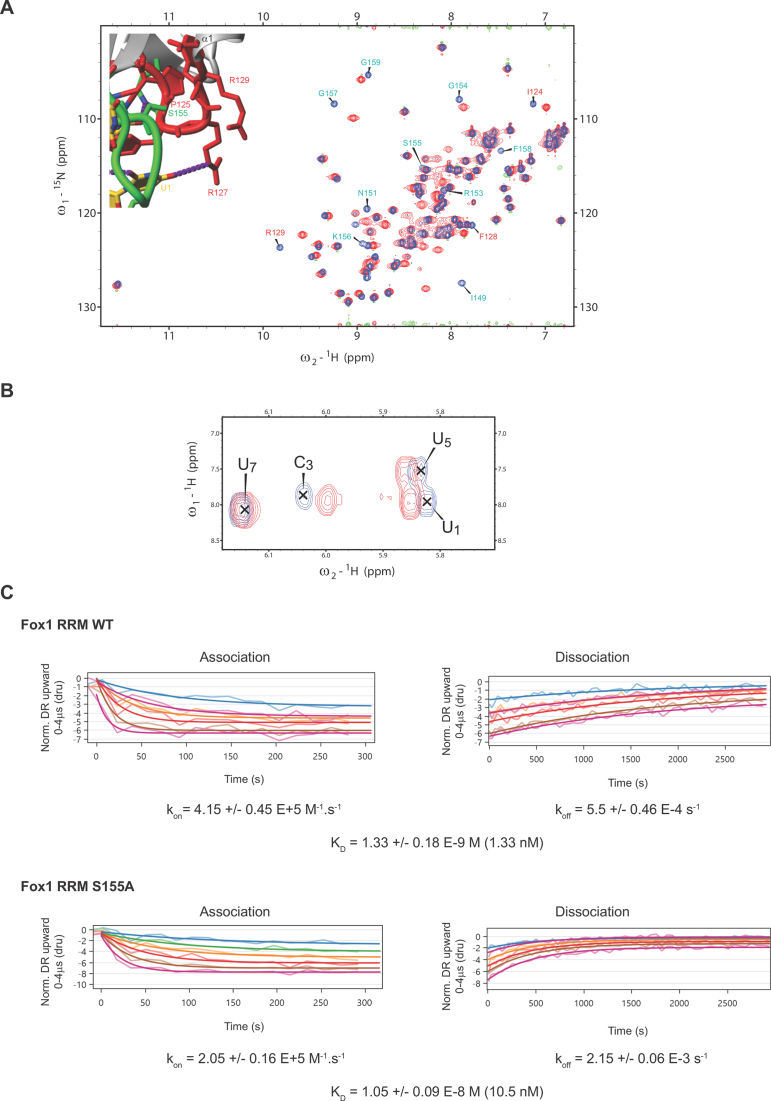
(**A**) Superimposition of ^1^H-^15^N HSQC spectra recorded with Fox-1 RRM WT (blue) and S155A (red). Those residues for which amide resonance differences were observed are labeled and shown on the protein/RNA structure (residues located in the β2/β3 and β1/α1 loops are in green and red, respectively). (**B**) Superimposition of ^1^H-^1^H TOCSY spectra recorded with Fox-1 RRM WT (blue) and S155A (red) both bound to the 5’- UGCAUGU-3’ RNA (at a 1:1 ratio). (**C**) Kinetic analysis of Fox-1 RRM WT and S155A proteins interacting with the 5’- UGCAUGU-3’ RNA using the switchSENSE technology. The raw data are superimposed by global exponential fits. The *k*_on_, *k*_off_ and *K*_D_ values are shown for each protein/RNA kinetics measurement.

The S155A mutation affected the NMR chemical shifts of amide resonances corresponding to residues surrounding the S155 residue which is part of the β2/β3 protein loop. At the same time, it had a strong impact on the chemical shifts of F128, R129 and I124 residues located in the β1/α1 protein loop which faces the S155 residue (Figure 4A). This observation was consistent with the elimination of a water molecule coordinated by the side-chain of S155 and the stabilization of the contact between these two protein loops. Further evidence of this water-mediated interaction was the presence of chemical shift differences for the H5-H6 cross-peaks of U_1_, C_3_ and U_5_ between the Fox WT-RNA and Fox S155A–RNA complexes (Figure 4B). To evaluate the importance of this water molecule for the recruitment of Fox-1 on RNA, we tested the effect of the S155A mutation on Fox-1 RRM affinity for the 5’-UGCAUGU-3’ RNA using switchSENSE technology (see ‘Materials and Methods’ section) (75,76). As illustrated in Figure 4C, with Fox-1 RRM WT, we obtained a *K*_D_ value of 1.33 nM, which was close to the value determined previously by Surface Plasmon Resonance (*K*_D_ of 1.09 nM) (4). We then tested the binding of the Fox-1 S155A variant to the same RNA and determined a *K*_D_ value of 10.5 nM (Figure 4C). This strong decrease in affinity (a factor of 7.9, i.e. ∼1.2 kcal/mol) can be explained by destabilization of the β2/β3 protein loop (involved in RNA binding) caused by the disruption of the S155 water-mediated interaction. This result is in excellent agreement with the TI calculations, which predicted an identical energy penalty for this mutation. Although such quantitative agreement between the TI computations and experiments is perhaps fortuitous, all methods support the importance of the hydration sites for both structural dynamics and the stability of the complex.

### Simulations of the Fox-1(S122A) system

A hydration site in the WT simulations mediated an interaction between the A_4_(O2΄) and S122(OG1) atoms (Figure 3, #C1). Since S122 could be actively contributing to the protein/RNA affinity, we mutated the S122 into alanine in order to abolish this hydration site.

In the first simulation (Fox-1(complex)_12_S122A), A_4_ showed increasing fluctuations with progressing simulation time. After 110 ns, it showed a pronounced movement away from the protein and permanently lost its intramolecular pairing with G_2_. It did not form any new stable interactions with the protein and fluctuated wildly for the rest of the 500 ns simulation. In the other two simulations (Fox-1(complex)_12_S122A_2 and Fox-1(complex)_14_S122A), A_4_ remained at its initial position but displayed larger thermal fluctuations compared with the WT simulations. These fluctuations were transferred to G_2_ via the G_2_/A_4_ intramolecular base pair and the F126 hydrophobic pocket (Supplementary Figure S8) was eventually completely disrupted.

While the simulations displayed variable behavior, they all showed perturbation of the protein/RNA interface when the S122 residue was mutated. Since the S122 side-chain does not form any other interactions, the hydration site between S122(OG1) and A_4_(O2΄) appears to be significant. Without it, the system becomes less constrained. In the first simulation, this caused A_4_ to move away from the structure. In the other two simulations, the increased thermal fluctuations eventually disrupted the F126 hydrophobic pocket. The TI free-energy calculation predicted a large free-energy penalization of 2.4 ± 0.2 kcal/mol for the mutant complex formation compared to the WT. Thus, the simulations predict a significant effect of this mutation on the protein affinity to RNA.

### Experiments on the Fox-1(S122A) system

**Figure 5.**
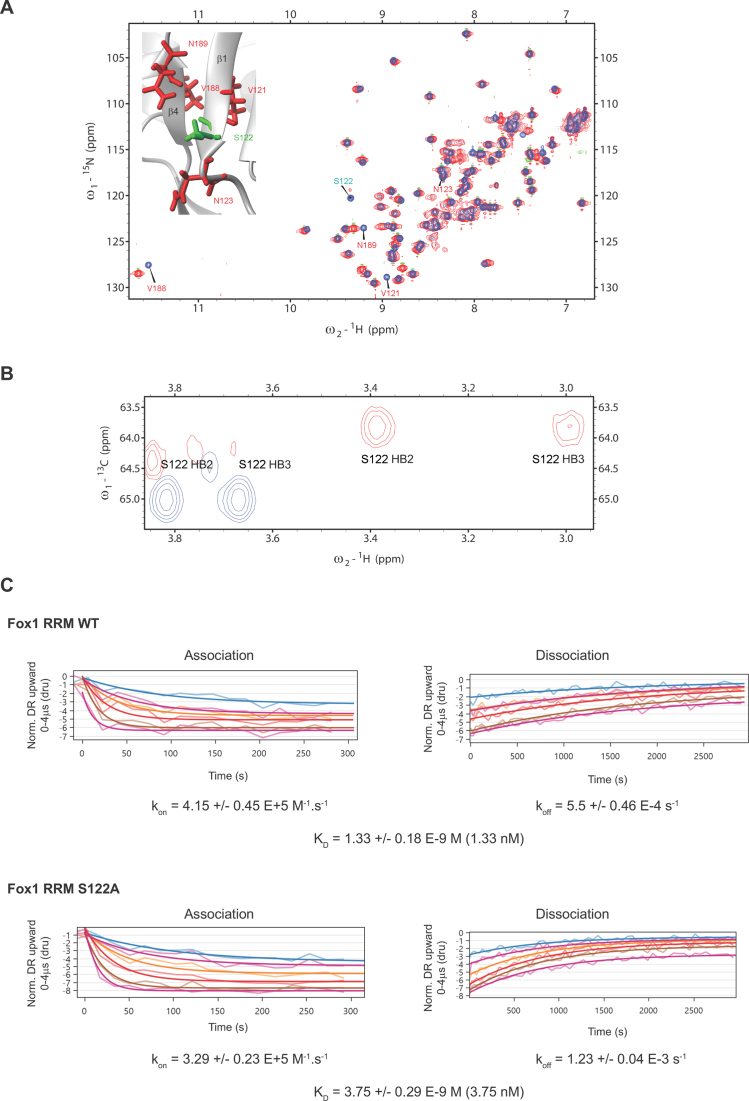
(**A**) Superimposition of ^1^H-^15^N HSQC spectra recorded with Fox-1 RRM WT (in blue) and S122A (in red). Those residues for which amide resonance differences were observed are labeled and shown on the protein/RNA structure. (**B**) Superimposition of ^1^H-^13^C HSQC spectra recorded with free Fox-1 RRM WT (blue) and its complex with the 5’-UGCAUGU-3’ RNA (red). (**C**) Kinetic analysis of Fox-1 RRM WT and S122A proteins interacting with the 5’-UGCAUGU-3’ RNA using the switchSENSE technology. The raw data are superimposed by global exponential fits. The *k*_on_, *k*_off_ and *K*_D_ values are shown for each protein/RNA kinetics measurement.

We first investigated the effect of the S122A mutation on the structure of Fox-1 RRM by recording ^1^H-^15^N HSQC spectra with the free form of the WT and mutated proteins. Only minor differences were observed, corresponding to chemical shift perturbations of amide resonances from residues surrounding S122 (Figure 5A). This effect was expected because the mutation of S122 modifies the electronic environment of the neighboring residues. In agreement with the involvement of a water molecule mediating an interaction between the A_4_ (O2΄) and S122(OG1) atoms, large chemical shift perturbations were observed upon RNA binding for S122 HB2 and HB3 resonances (Figure 5B). The NMR data are thus consistent with presence of a hydration site predicted by MD in the WT system.

We then tested the importance of this water-mediated interaction using the strategy described above for the S155A mutant and performed kinetics measurements in the presence of Fox-1 S122A and the 5’-UGCAUGU-3’ containing ssRNA. We detected only a slight decrease in affinity between the WT protein (*K*_D_ value of 1.33 nM) and the S122A mutant (*K*_D_ value of 3.75 nM) (Figure 5C) complexes. Although this result is consistent with the involvement of S122 in a water-mediated interaction with RNA, the effect on Fox-1 affinity (a factor of 2.8, i.e. ∼0.6 kcal/mol) is considerably smaller than the ∼2.4 kcal/mol free-energy prediction by the TI calculations. The computational method overestimates the free-energy effect. We state possible reasons for this in the ‘Discussion’ section below.

## DISCUSSION

We have presented a newly determined X-ray structure of the free RRM domain of the human Fox-1 protein, a well-known member of the Fox-1 family. The structure has been refined to a high resolution of 1.8 Å and contains six independent protein subunits in the asymmetric unit (Table 1). We have used this new structure, along with earlier NMR structure of the Fox-1 RRM protein/RNA complex (4), as a basis for MD simulations in explicit solvent. In our study, we focused on an analysis of the hydration sites in MD simulations and compared them with the solvation shell seen in the X-ray structure of the free Fox-1 RRM. Finally, we used targeted protein mutagenesis and NMR spectroscopy to analyze the potential role of two of the hydration sites on protein/RNA binding.

### Highly populated hydration sites—an excellent agreement between the X-ray and MD simulations data

The X-ray structure and MD simulations have shown superb agreement in the location of the hydration sites (defined by the identity of the coordinating solute atoms). We have shown that the hydration sites that are present within all six protein molecules of the X-ray structure (Table 3, hydration sites #1, #2, #3, #4 and #5) are also prominent in the MD simulations with water residency times greater than a nanosecond (Table 4); MD is thus clearly able to reproduce the experimental hydration sites. In addition, the MD simulations were able to predict similar hydration sites in the Fox-1 protein/RNA complex (starting from an NMR structure with no explicit water molecules). Thus, explicit-solvent MD can be used to predict hydration sites with a high degree of confidence in NMR structures where they are often not experimentally detectable.

The most salient hydration site appeared near the S155 residue where it stabilized the relative positions of the β1/α1 and β2/β3 loops toward each other (site #2 in Tables 3 and 4). The site had very long water-binding times in the complex in the range of hundreds of nanoseconds; by comparison, water residency times in the simplest hydration sites on the surface of biomolecules are in the sub-nanosecond range (25,26,37,42,61). The long-residency time of the S155 hydration site was due to the two protein loops obstructing exchanges with the solvent and three protein residues coordinating the water molecule. The water binding times were reduced in the simulations of the free Fox-1 RRM, suggesting that the bound RNA indirectly communicates with this hydration site (Table 4). It is likely that the RNA binding somewhat rigidifies the proximal protein moieties; similar effects have been reported for hydration sites in proteins (74). The S155 hydration site may be important for the structure of the RRM domain in general. Our database search suggests that the serine 155 residue is structurally conserved within a number of different RRM-domain-containing proteins (e.g. TDP-43 RRM1, UP1, Sup-12, hnRNP G, U2AF65, PABP, HuR, Musashi1, Tra2-beta, CUGBP1 and RNA15) (7,77–87) and the coordinated water molecule is present in multiple X-ray determined structures. We also observed it as a long-residency hydration site in the MD simulations of the CUGBP1 RRM3 system (unpublished data).

Another significant hydration site was identified near the H120 residue which stacks with the U_5_ base in the complex (Figure 3, #1). Even in the free protein, the coordination of the water molecule restricted the movements of the histidine side-chain and stabilized it in the conformation found in the protein/RNA complex. We hypothesize that it may facilitate RNA binding by increasing the chance of successful conformational capture by the RNA molecule.

The simulations also predict a number of hydration sites that are exclusive to the protein/RNA complex as the waters either bridge the protein/RNA atoms or involve the RNA atoms only. Most significantly, we show that the A_4_ nucleotide, which does not form any direct H-bonds with the protein, is involved in several water-mediated interactions (Supplementary Figure S7). Another example is the G_2_ nucleotide where the water molecules bridge the guanine O6 carbonyl atom and the β1/α1 loop N-H amides (Supplementary Figure S6, #C4). Finally, we predict an altered hydration pattern of R118 as a result of the RNA binding (Figure 3, #5). Altogether, the simulations suggest that water molecules are playing a key role in the formation of the non-canonical A_4_/G_2_ base-pair found in the Fox-1/RNA complex (4).

### Can observed hydration sites be used to predict RNA binding?

A tempting application of our study of hydration sites in the free Fox-1 RRM would be a prediction of the binding site of the target RNA molecule. In this study, we had available the experimental structures of both the free Fox-1 RRM and its protein/RNA complex. However, there are a number of free RRM protein structures in the database for which the atomic structures with their target RNA are unknown and difficult to determine experimentally due to, e.g. low binding affinity (35). Previous studies have suggested that having knowledge concerning the hydration shell of a biomolecule may be used to predict the structural properties of its interaction with a ligand (88,89). Unfortunately, we were unable to utilize our simulation data of the free Fox-1 RRM for a similar goal, i.e. predicting the RNA binding site. One of the obstacles was the simulation fluctuations of the surface amino acid side-chains, which resulted in a high degree of noise in the data. To overcome this, we applied positional restraints to the solute atoms to temporarily suppress the fluctuations in some simulations (see Table 2). However, we did not find any clear way of identifying which of the many hydration sites correspond to the actual RNA binding site. Note that we would face the same problem also when using the X-ray hydration pattern, which, in addition, is visibly heterogeneous, when comparing the six crystallographically independent units. Thus, although we agree that knowledge of the hydration sites could be used to guess binding modes in some systems, the practical application of such knowledge may be less straightforward than was earlier suggested (88,89).

### The Fox-1 S155A and S122A mutant studies

We have tried cross-validation of the MD predictions by further NMR and biochemical experiments. This can be straightforwardly attempted when the water molecule is coordinated by an amino acid side-chain; then, a single-point substitution for similarly sized side-chain can be used to disrupt the hydration site and so allow its impact on the mutated system to be studied. Thus, our additional experiments included the hydration sites near residues S155 and S122, which were predicted by the MD simulations (see above and Figure 3, sites labeled as #2 and #C1). In MD, the hydration site near S155 is a long-residency hydration site with water binding times in the hundreds of nanoseconds. It is seen in all molecules of the X-ray structure of the free Fox-1 RRM. The S122 hydration site is predicted to form at the protein/RNA interface and its water molecules exchange more rapidly with residency times in the nanosecond to tens of nanoseconds range.

Our NMR experiments investigating chemical shift changes upon introducing a mutation designed to eliminate the water coordination site near the S155 residue showed strong chemical shift changes in the residues that interact with the water molecule. SwitchSENSE measurements indicated an energy penalty of 1.2 kcal/mol (Figure 4) for the complex formation. These experimental results were in excellent agreement with the TI free-energy calculations, which predicted an identical energy penalty for this mutation. Even though the S155 hydration site is coordinated exclusively by protein atoms, it stabilizes the β1/α1 and β2/β3 protein loops which contain many residues involved in the protein/RNA interface. Thus, we suggest that the abolishment of this hydration site lowers the RNA binding affinity via allosteric effects.

The experiments also provide evidence to support the presence of the predicted water molecule near the S122 residue (Figure 5). The S122A mutation penalizes the formation of the protein/RNA complex by 0.6 kcal/mol. In this particular case the TI computations rather significantly overestimate the measurement, as the computed free-energy effect is 2.4 kcal/mol. The reason for this difference is not clear; nevertheless, the TI free-energy method is very sensitive to force-field and sampling issues (90), so it is possible that in this particular case the theoretical protocol was not robust enough to provide a quantitative picture of the structural/energy response to the substitution. This can be due to number of factors. In the computations, the mutation is done on an existing WT complex, allowing us to observe the *destabilization* of the mutated complex on a sub-microsecond time-scale after a hypothetical amino acid exchange inside the WT complex. If there are structural relaxations that would require a longer time-scale to equilibrate the system after the exchange, they would remain undetectable by the simulation method. By contrast, in the experiment, the mutation is done on a lone protein before the complex formation. It is thus possible that in this particular case the simulation time-scale was not long enough for it to capture the full impact of the S122A substitution, either in the isolated protein or in the complex (or in both).

Alternatively, although not apparent from the WT simulations, the performance of the biomolecular force field might not be completely accurate in this specific case, resulting in an imperfect prediction of the S122A mutation effect. Specifically, the force field could be incorrectly overpopulating the protein/RNA interface conformation in which the water-mediated site near the S122 exists, whereas it has only a minor population in the experiment (as suggested by the lower energy penalty measured). This did not affect the WT simulations because the conformation is structurally realistic, albeit overestimated in the simulation ensemble. However, it may bias the prediction of the S122A mutation effect. The result of the free-energy computation for the S122A mutation is in any case somewhat unsatisfactory and confirms that quantitative free-energy computations on biomolecular systems remain challenging; the MD methodology is considerably more reliable in description of hydration structural dynamics than in free energy computations (20,21,24).

### The ff12SB protein force field provides the best performance in MD

Our earlier study of the Fox-1 and SRSF1 RRM protein/RNA complexes (56), focusing on the protein/RNA interface dynamics, indicated that the ff12SB and ff14SB AMBER protein force fields (51) are superior to the older ff99SB. The present analysis indicates that the ff12SB reproduces the internal structure of the Fox-1 RRM (which is unrelated to the protein/RNA interface) better than the ff14SB. Specifically, with the ff99SB and ff14SB we observe somewhat unsettling behavior of some of the phenylalanine side-chains which are part of the hydrophobic core of the protein. Excessive movements of these residues sometimes led to pronounced distortions of the protein α2 helix in the MD simulations (Supplementary Figure S9). We eventually traced this behavior to the phenylalanine χ_2_ dihedral term, which has an increased energy barrier of the aromatic ring rotation in the ff12SB force field. The energy barrier is more than twice that of ff99SB. Our recent simulation benchmark study of six protein/RNA complexes (55) indeed suggested that the reparametrization of the phenylalanine and tyrosine side-chain dihedrals was a visible improvement brought by the ff12SB protein force field. However, it appears that the ff14SB force field (at least in its current implementation) returned to the ff99SB in these particular parameters (Supplementary Figure S10). This results in fast rotations of the phenylalanine aromatic rings and the eventual structural perturbations observed in our simulations. Considering that ff12SB is a preliminary version of ff14SB containing most of the ff14SB improvements (51), this force-field difference is somewhat surprising. For the RNA, we use the χ_OL3_ force field (current AMBER default for RNA) which provides a satisfactory description of the RNA molecule within the protein/RNA complex systems (55,56).

### Comparison of the X-ray and NMR structures reveals a structural difference in the β3/α2 loop

The structure of the free Fox-1 RRM revealed by our X-ray structure is very close to the structure of the protein/RNA complex shown by NMR (4). Nevertheless, there is a clear difference in the β3/α2 loop region of the protein. In essence, the area is more opened-up in the NMR structure and shows different interactions (see above and Figure 1). However, as reported above, the difference may potentially be due to an incorrect assignment of the F163 side-chain χ_1_ dihedral angle in the NMR structure of the protein/RNA complex (4); it almost certainly is not related to the RNA binding. This was indicated by the MD simulations of the free Fox-1 RRM obtained by removal of the RNA from the NMR structure of the protein/RNA complex where the β3/α2 loop structure did not show any signs of converging toward the X-ray structure arrangement. At the same time, the MD simulations were unable to spontaneously fix the potentially incorrect F163 side-chain dihedral as this would require at least a partial unfolding of the protein. Such a conformational change was likely beyond the microsecond simulation time-scale. Since this region is situated far from the protein–RNA interface, no further efforts were made to investigate the β3/α2 loop conformation in the solution structure.

## CONCLUSION

Our study suggests that explicit solvent atomistic simulations are sufficiently robust to predict, with a high degree of confidence, the hydration sites in the NMR-derived structures where information concerning the solvent is generally difficult to obtain. MD can also be used to interpret the biomolecule hydration patterns observed in the X-ray structures and highlight the most stably occupied hydration sites. We demonstrate this by combining the hydration data from the newly resolved X-ray structure of the free Fox-1 RRM, the MD simulations of this structure and of the RRM complexed with its target RNA, as well as NMR measurements, switchSENSE experiments and simulations with Fox-1 mutants. The data show a good degree of agreement between the methods and suggest that the analysis of specific hydration is one of the more powerful applications of the simulation methodology. The structural dynamics hydration data obtained from MD simulations appears to be very reliable. The free-energy calculations of changes caused by amino acid substitutions abolishing the hydration sites are less reliable due to sampling limitations and possibly due to the high sensitivity to the precision of the force field. Nevertheless, with proper acknowledgment of the limitations, the MD simulations can be routinely used as a complement of the X-ray and NMR structural studies to capture the hydration patterns in the protein/RNA complexes. In this regard, our combined experimental and theoretical study has allowed us to gain insights into two long-lived water molecules that play a role in the sequence-specific recognition of the RRM of Fox-1 for RNA. Furthermore, one of these hydration sites is conserved in several other RRM domains.

## ACCESSION NUMBER

Coordinates and structure factors are deposited in the RCSB protein data bank (PDB) with the accession number 4zka.

## Supplementary Material

Supplementary Data
