# Supplementary material for: Structural study of the Fox-1 RRM protein hydration reveals a role for key water molecules in RRM-RNA recognition
Source: Nucleic Acids Res. 2017 May 13;45(13):8046–63. doi: 10.1093/nar/gkx418 (PMC5737849; doi:10.1093/nar/gkx418)
Supplement: Supplementary Data [file gkx418_supp.docx]

**Structural study of the Fox-1 RRM protein hydration reveals a role for key water molecules in RRM-RNA recognition**

Miroslav Krepl^1,2^*, Markus Blatter^3,4^, Antoine Cléry^3^, Fred F. Damberger^3^, Frédéric H.T. Allain^3^*, and Jiri Sponer^1,2^*

^1^Institute of Biophysics, Academy of Sciences of the Czech Republic, Kralovopolska 135, 612 65 Brno, Czech Republic

^2^Regional Centre of Advanced Technologies and Materials, Department of Physical Chemistry, Faculty of Science, Palacky University Olomouc, 17. listopadu 12, 771 46 Olomouc, Czech Republic

^3^Institute of Molecular Biology and Biophysics, Department of Biology, ETH Zurich, CH-8093 Zurich, Switzerland

^4^Present address: Global Discovery Chemistry, Novartis Institute for BioMedical Research, Basel CH-4002, Switzerland

* To whom correspondence should be addressed. Tel: +420 541 517 266; Email: [miroslav.krepl@upol.cz](mailto:miroslav.krepl@upol.cz); Correspondence may also be addressed to Frederic H.T. Allain. Tel: +41 44 633 39 40; Fax: +41 44 633 12 94; Email: [allain@mol.biol.ethz.ch](mailto:allain@mol.biol.ethz.ch) and to Jiri Sponer. Tel: +420 549 49 8219; Fax: +420 541 212 179; Email: [sponer@ncbr.muni.cz](mailto:sponer@ncbr.muni.cz)

# Supporting Information

**Crystallographic data of the water molecules present in all six protein molecules of the asymmetric unit.** Ammonium ion is the only buffer component which cannot be distinguished from water molecules. Therefore, all refined water molecules could in principal also be ammonia molecules. However, the ammonia content in the buffer was less than 20 mM. In addition, the discussed water sites include many hydrogen bond donors which make confusion with ammonium ions unlikely. The discussed water molecules mostly possess occupancies close or equal to 1 (Table S1). A notable exception is the water 1 which has somewhat lover occupancy values (0.54 and 0.63) for two out of six protein molecules.

Water site 1 is in particular interesting because the involved H120 and N189 side chains are at the RNA interface and asparagine or aspartate are highly abundant at this position among other RRMs. This β1 binding pocket accommodates a uracil base in the case of Fox-1 RRM. However, in many other RRM protein/RNA complexes, this prominent pocket is occupied by a cytosine which then acts as a hydrogen bond donor to the side chain of the mentioned asparagine or aspartate. If further studies show that this water is generally present, it will be interesting to investigate its role in entropic changes when the water has to be released upon RNA binding of a cytosine base. Note that differential changes in hydration upon RRM protein/RNA complex formation were recently reported in *Samatanga et al., 2017, Nucleic acids research, DOI: 10.1093/nar/gkx136*.

**The Fox-1(free*) simulations and the behavior of the F163 residue.** As noted in the main text, the β3/α2 loop in the NMR structure adopts a different arrangement compared to the X-ray structure, and it is likely that the F163 orientation in the NMR (and thus starting) structure has been incorrectly refined (see the main text). Despite using long simulations (the longest Fox-1(free*) simulation being 7 μs long), the conformation of the β3/α2 loop region seen in the protein/RNA complex structure was fully maintained in all the Fox-1(free*) simulations, including its signature interactions. In conclusion, the Fox-1(free) and Fox-1(free*) simulations converged to the same ensemble behavior for those parts of the protein where the X-ray and NMR structures agreed, but were not long enough to eliminate the initial-structure effects for those parts of the system (i.e., the β3/α2 loop region) that differed in the experimental structures.

**Phenylalanine flips in different force-field versions.** We observed frequent flips of several phenylalanine χ_2_ dihedrals (CA-CB-CG-CD, i.e., the rotation of the aromatic ring) between gauche(+) and gauche(-) in the ff99SB and ff14SB simulations. While both of the χ_2_ rotamers are chemically equivalent for phenylalanine, the (to our opinion excessive) rotations of the rings in the simulations were disturbing the surrounding regions, especially in the tightly packed hydrophobic core of the protein. This behavior was entirely absent with the ff12SB protein force field. It could be attributed to (in our opinion) inferior description of aromatic ring rotation in the ff99SB force field which was fixed with the ff12SB reparametrization but reoccurred in the current implementation of the ff14SB.

**Hydration sites related to the β3/α2 protein loop in the Fox-1(complex) simulations.** There were two groups of unique hydration sites in the simulations of the protein/RNA complex. The first group was related to the X-ray vs. NMR structure difference in the β3/α2 protein loop region (see main text Figure 1 and 2) which, however, may be an error of the NMR structure. In simulations, this structural difference persisted even upon removal of the RNA and therefore, these hydration sites were present also in the Fox-1(free*) simulations. The two main hydration sites related to the β3/α2 protein loop had waters coordinated by E164(*sc*) and S166(O) atoms and by K142(O) and F163(O) atoms. The two sites were inter-connected via a third water molecule interacting with the F163(O) atom and the water molecule coordinated by the E164(*sc*) and S166(O) atoms (Figure S6, sites #C5 and #C6). Yet another hydration site was composed of periodically dissolving salt-bridge formed by E164(*sc*), D170(*sc*), and R173(*sc*) atoms (Figure S6, site #C7).

The second group of hydration sites was directly related to the RNA binding. They were absent in the Fox-1(free*) simulations and are described in the main text.

# Supporting Information Tables

Table S1. List of the five water molecules which are present in all six protein molecules of the asymmetric unit of the free Fox-1 RRM X-ray structure (PDB: 4zka). The water residue number (res) in the respective protein molecule, the values for occupancy (occ), and temperature factor (bf) are shown.

| **Water** | **1** | | | **2** | | | **3** | | | **4** | | | **5** | | |
| --- | --- | --- | --- | --- | --- | --- | --- | --- | --- | --- | --- | --- | --- | --- | --- |
| **Value** | **res** | **occ** | **bf** | **res** | **occ** | **bf** | **res** | **occ** | **bf** | **res** | **occ** | **bf** | **res** | **occ** | **bf** |
| **Molecule A** | 317 | 0.75 | 15.49 | 312 | 1.00 | 13.35 | 319 | 1.00 | 18.02 | 321 | 1.00 | 33.922 | 320 | 0.89 | 26.53 |
| **Molecule B** | 323 | 0.54 | 11.88 | 322 | 1.00 | 12.19 | 335 | 0.97 | 16.36 | 315 | 1.00 | 27.97 | 309 | 1.00 | 31.42 |
| **Molecule C** | 306 | 0.86 | 31.54 | 314 | 1.00 | 34.78 | 305 | 0.87 | 20.48 | 327 | 0.82 | 35.57 | 302 | 1.00 | 40.08 |
| **Molecule D** | 333 | 1.00 | 20.87 | 311 | 1.00 | 13.09 | 324 | 1.00 | 16.87 | 320 | 1.00 | 28.37 | 323 | 1.00 | 21.86 |
| **Molecule E** | 317 | 1.00 | 26.00 | 318 | 1.00 | 16.82 | 335 | 1.00 | 15.54 | 327 | 1.00 | 30.92 | 307 | 0.84 | 25.42 |
| **Molecule F** | 331 | 0.63 | 19.89 | 313 | 1.00 | 12.24 | 312 | 1.00 | 28.57 | 335 | 1.00 | 29.66 | 305 | 1.00 | 34.44 |
| **Electron Density map for waters in Molecule D** | **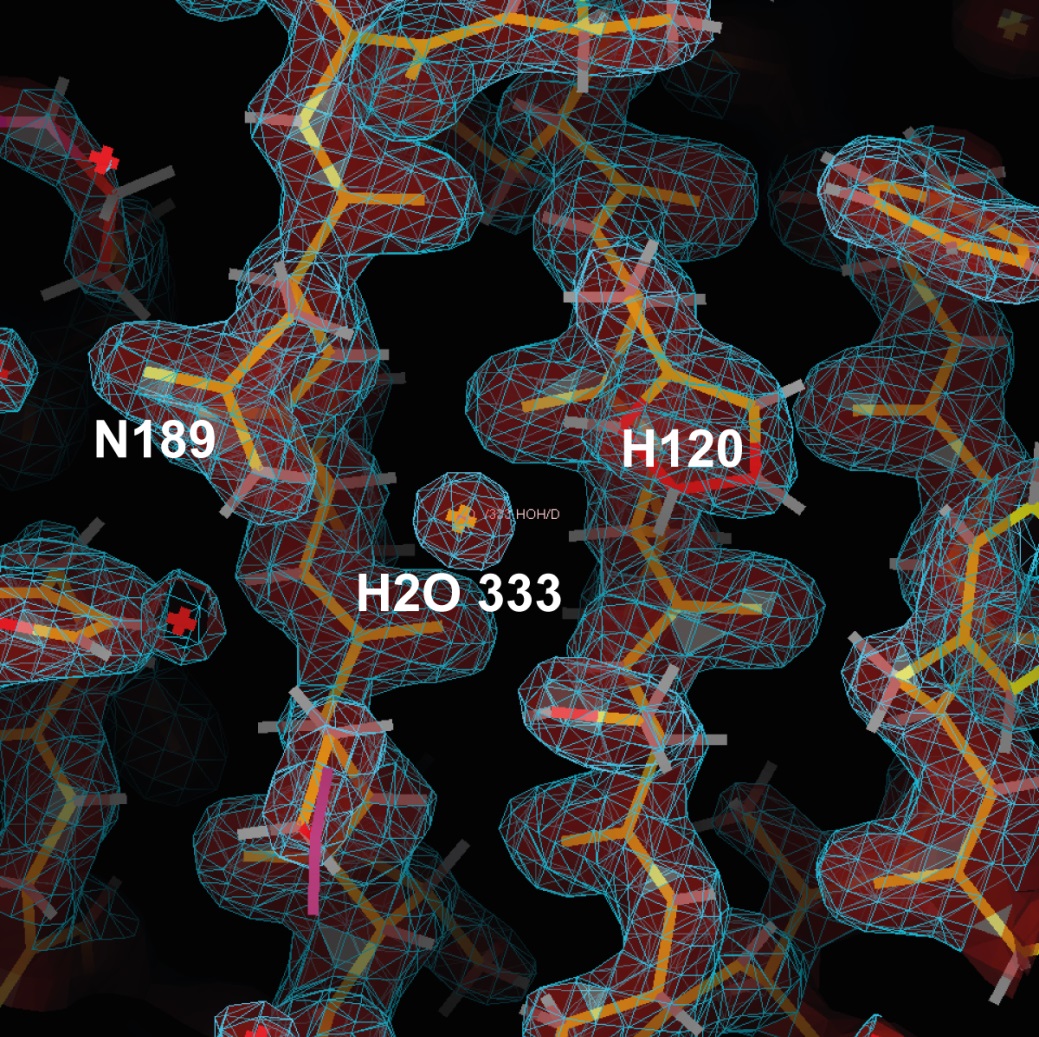** | | | **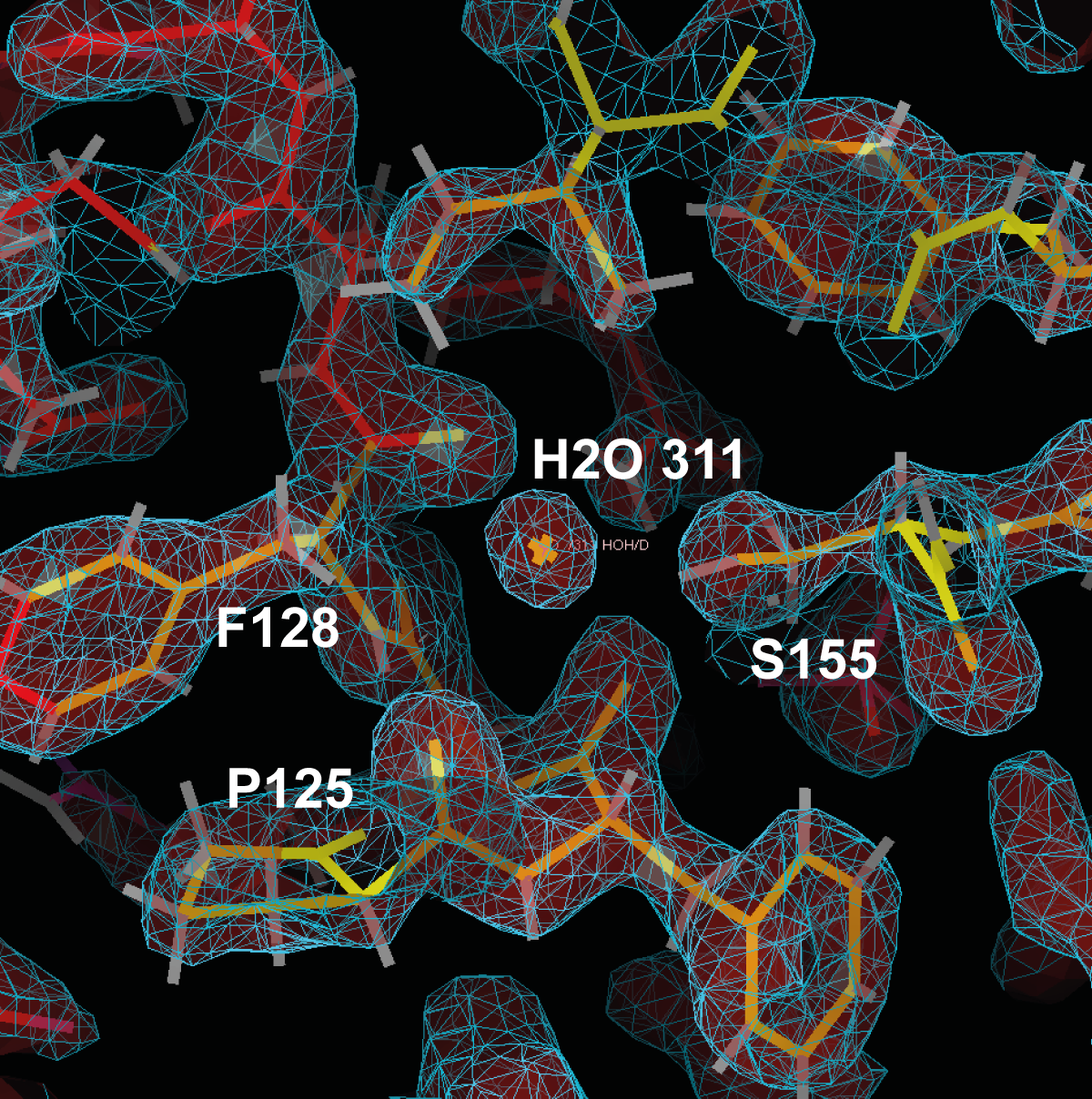** | | | **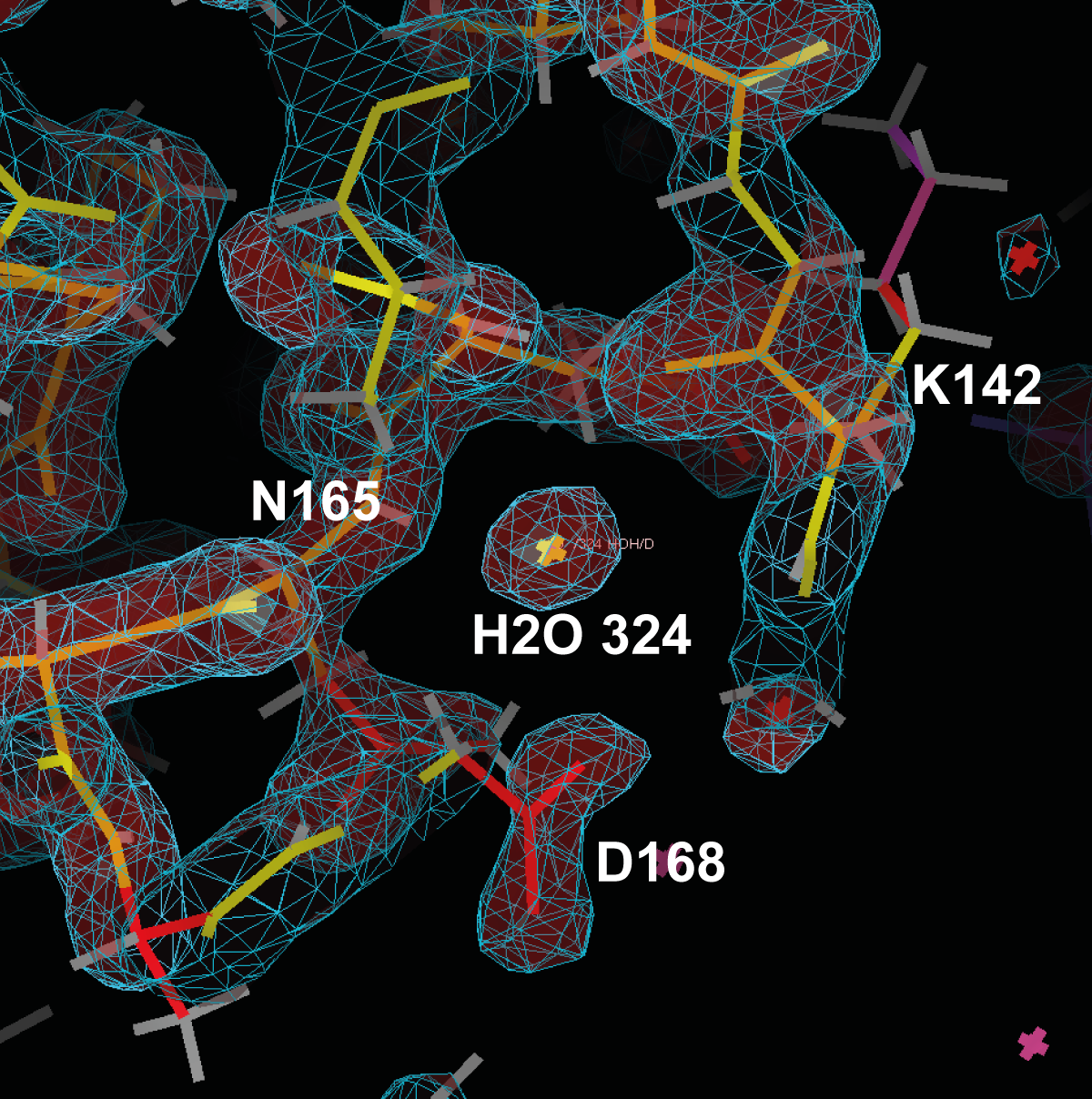** | | | **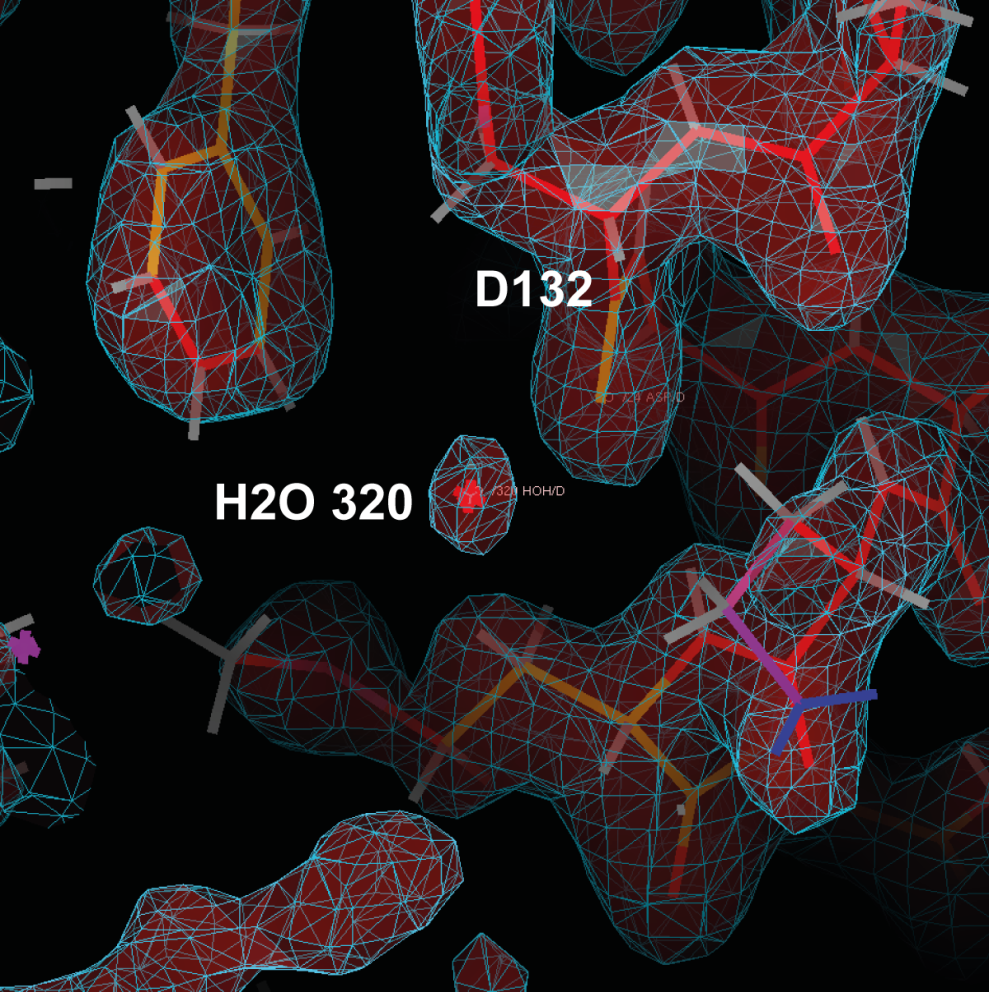** | | | **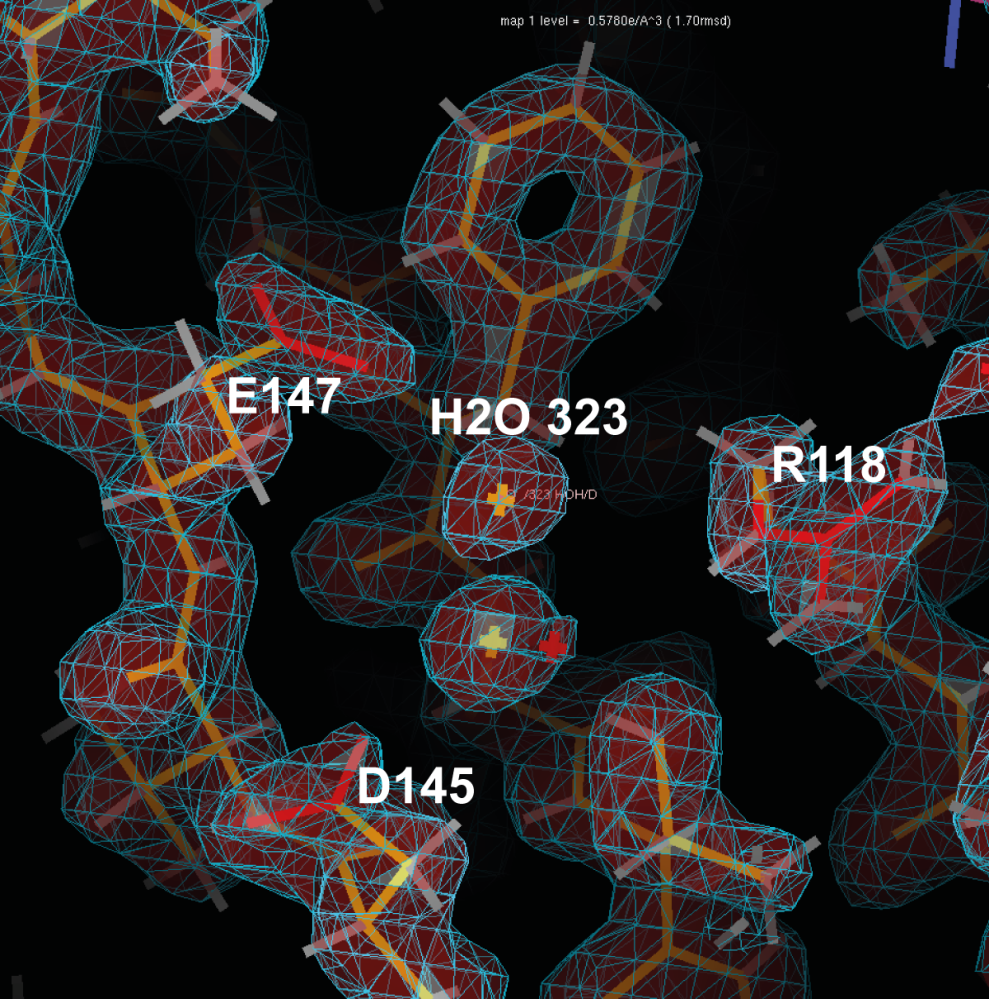** | | |

# Supporting Information Figures

**
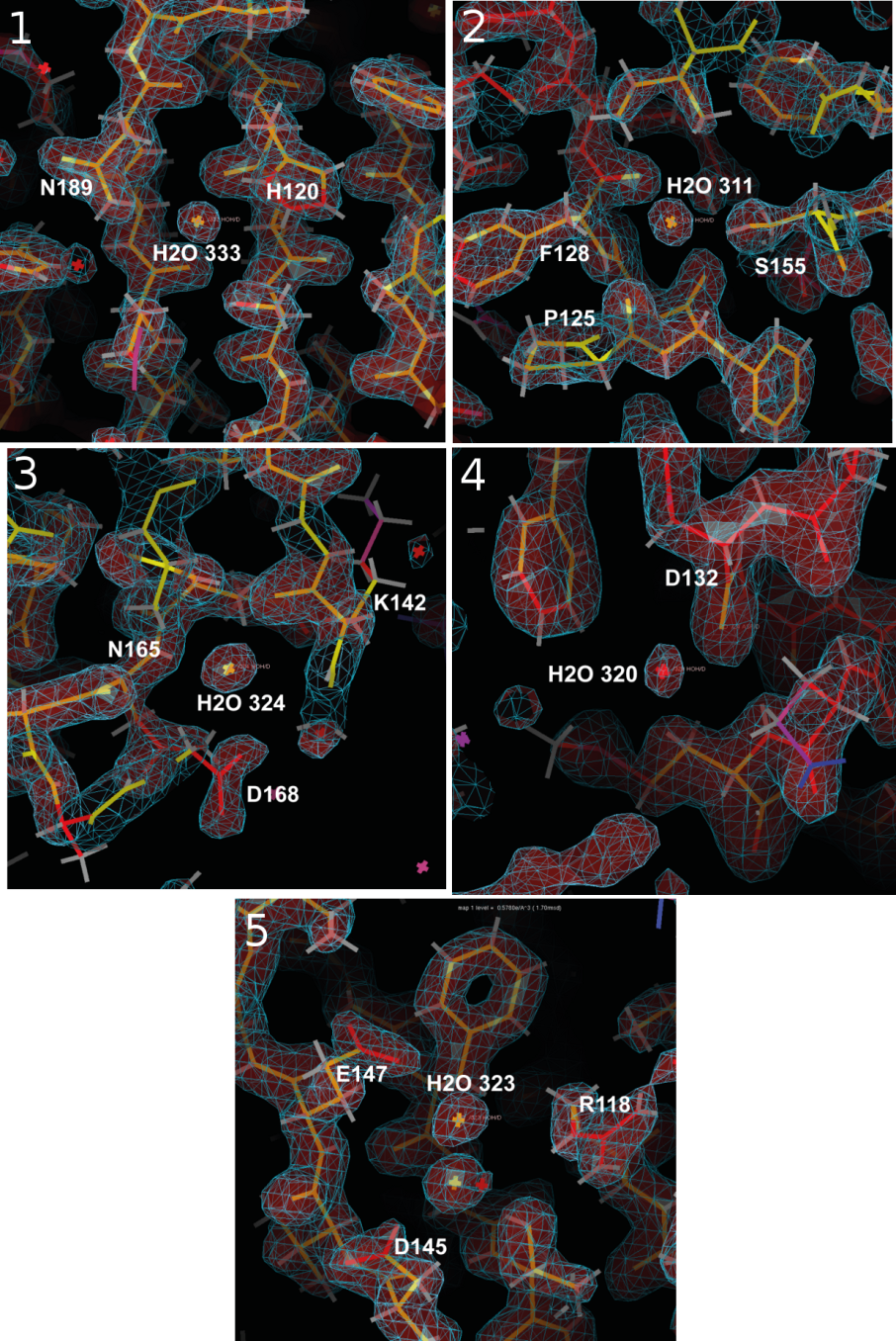
**

Figure S1. Visualization of the electron density maps of the discussed water sites 1 to 5 (see the main text Table 3). The bonds are colored by temperature factor. The interacting amino acids are labeled and the water PDB number (chain D in 4zka) is given.


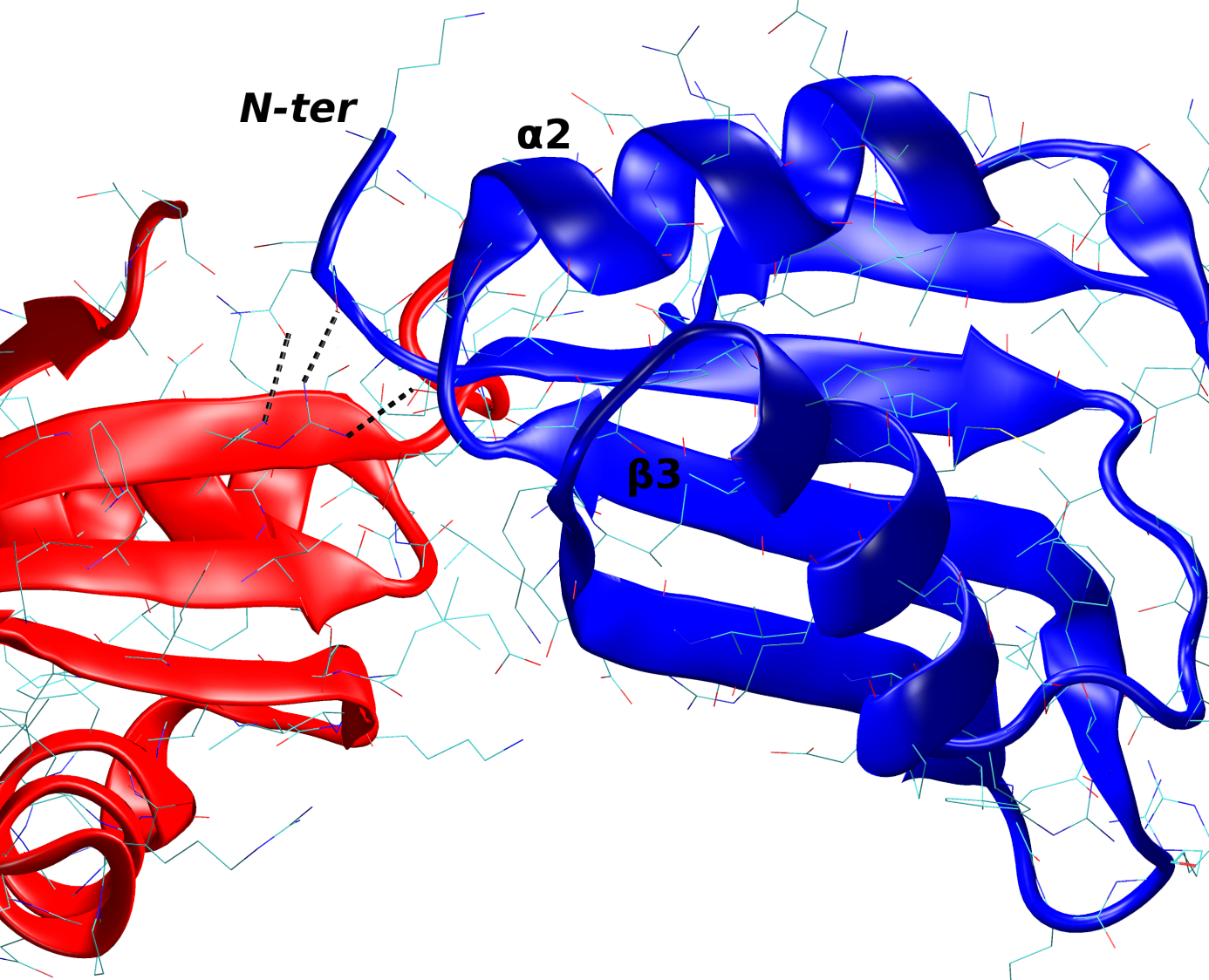


Figure S2. The crystal packing interactions of the β3/α2 segment in the X-ray structure of the free Fox-1 protein. The β3 sheet, α2 helix, and the N-terminal chain end are labeled. The protein chain in the primary crystal cell is in blue. The protein chain in the neighboring crystal cell is in red. The crystal packing H-bond interactions between them are indicated by black dotted lines.


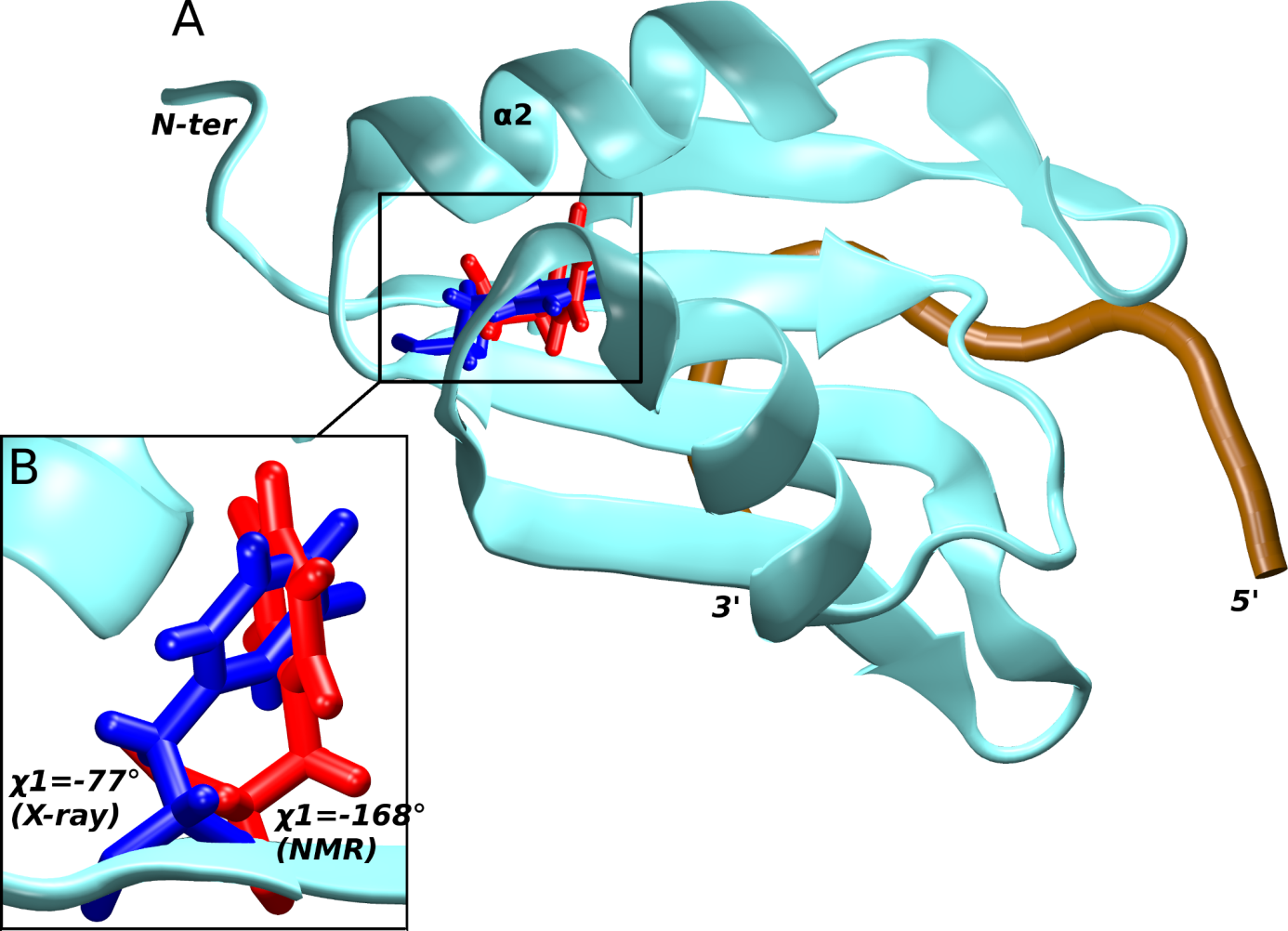


Figure S3. (A) Structure of the Fox-1 RRM showing the different conformation of the F163 side-chain in the X-ray (blue) and NMR (red) structures, respectively. Helix α2 and chain termini are labeled. The position of the RNA backbone in a formed complex is traced in brown. (B) Detail of the F163 residue. In the X-ray structure, its χ1 side chain dihedral is in the *gauche-* region while in NMR, it is in *trans*. Note that F163 is sufficiently separated from the protein/RNA interface and thus eventual error in the F163 χ1 side chain dihedral should not affect any conclusions of our paper.


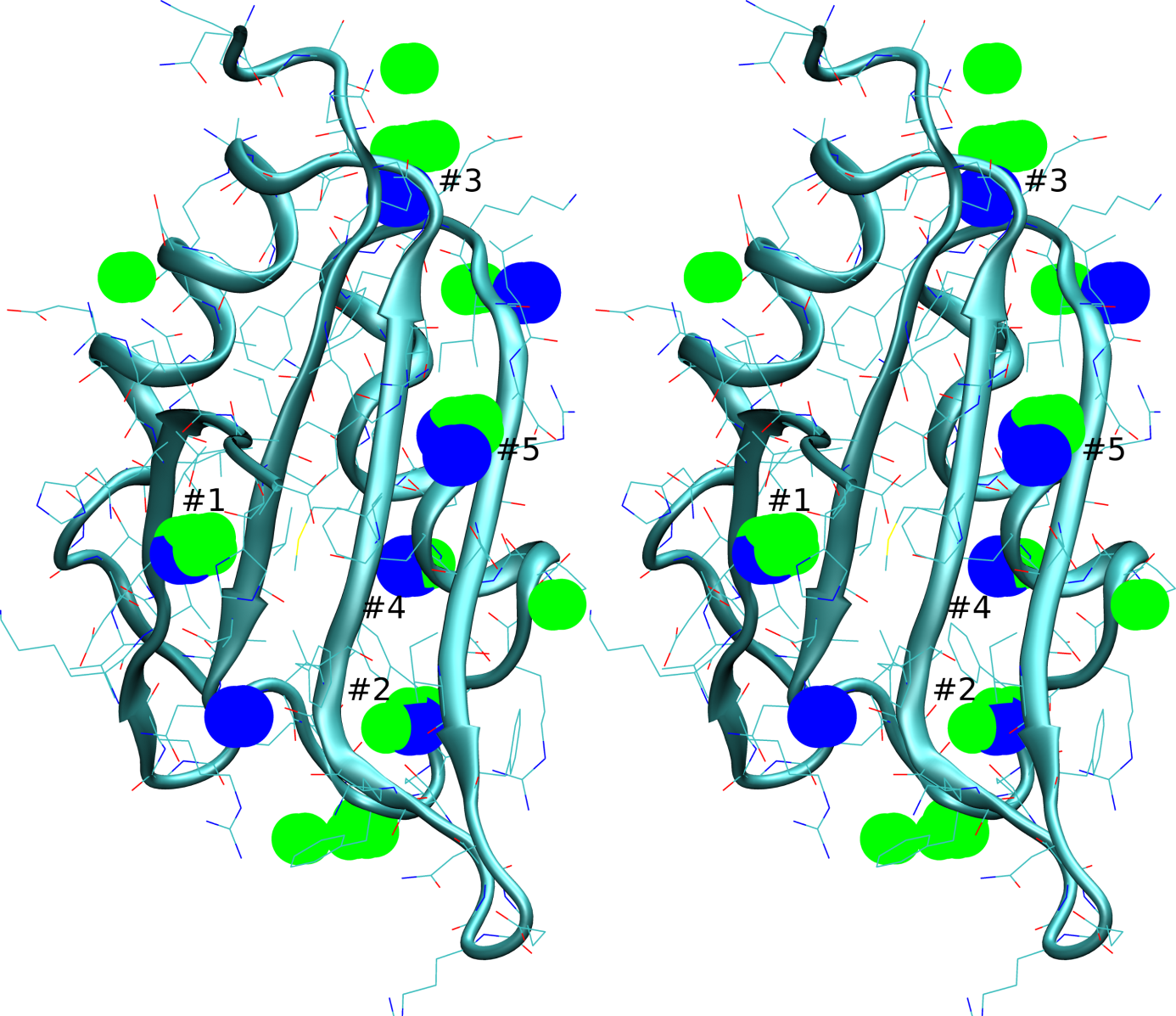


Figure S4. A stereoscopic view of the representation of seven highest grid water density points around the Fox-1 RRM in the X-ray structure of the free protein (blue spheres) and the Fox-1(free)_12_1 simulation (green spheres). The overlapping density points, corresponding to specific hydration sites, are labeled as defined in main text Table 3.


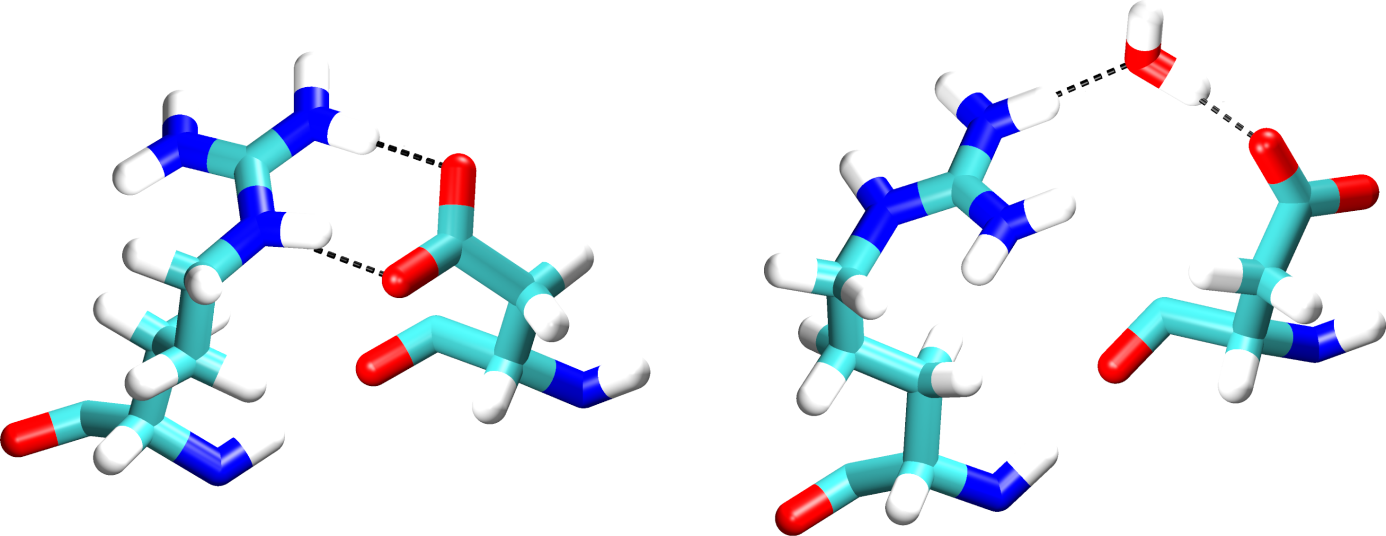


Figure S5. An example of a typical “transient salt-bridge” commonly seen in the MD simulations of the Fox-1 RRM. The interaction between arginine and aspartate side-chains periodically fluctuated between direct (left) and water-mediated (right) interaction. The black dotted lines indicate the H-bonds.


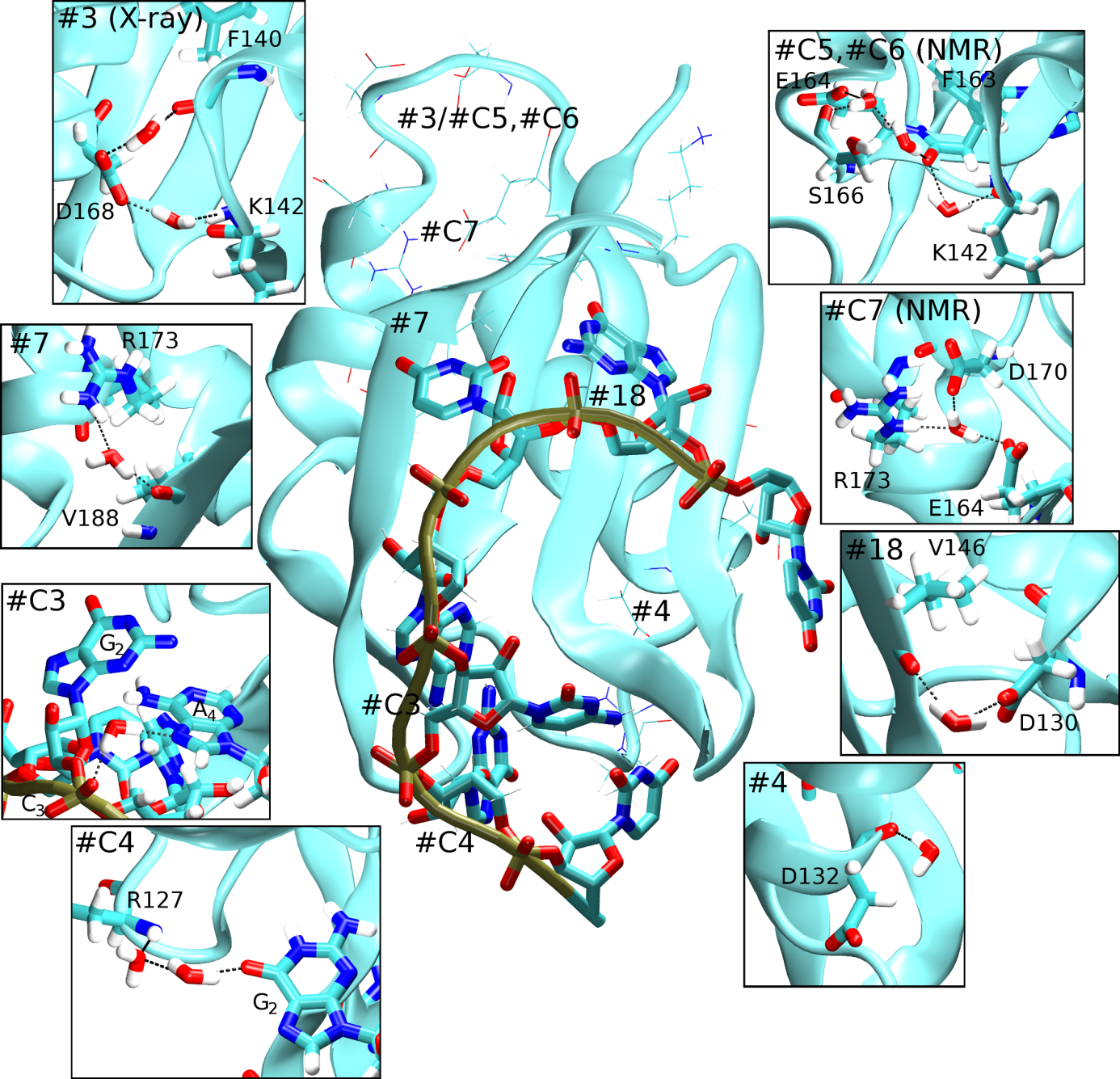


Figure S6. Selected hydration sites observed in the simulations of the Fox-1 RRM system. The inset figures show details of the individual hydration sites with the water coordination indicated by dotted black lines and the coordinating residues labeled. The hydration sites are labeled as defined in main text Tables 3 and 4. Sites #4, #7, and #18 were seen in simulations of both the free protein and the protein/RNA complex. Site #3 was seen only in the X-ray structure of the free Fox-1 RRM and in the Fox-1(free) simulations. Sites #C5/#C6 and #C7 were exclusive to the Fox-1(free*) and Fox-1(complex) simulations, both based on the NMR structure of the protein/RNA complex. Site #C3 was coordinated by the RNA atoms and therefore exclusive to the Fox-1(complex) simulations. Site #C4 involved a water bridge to an RNA atom via a hydration site that already exists in the free protein.


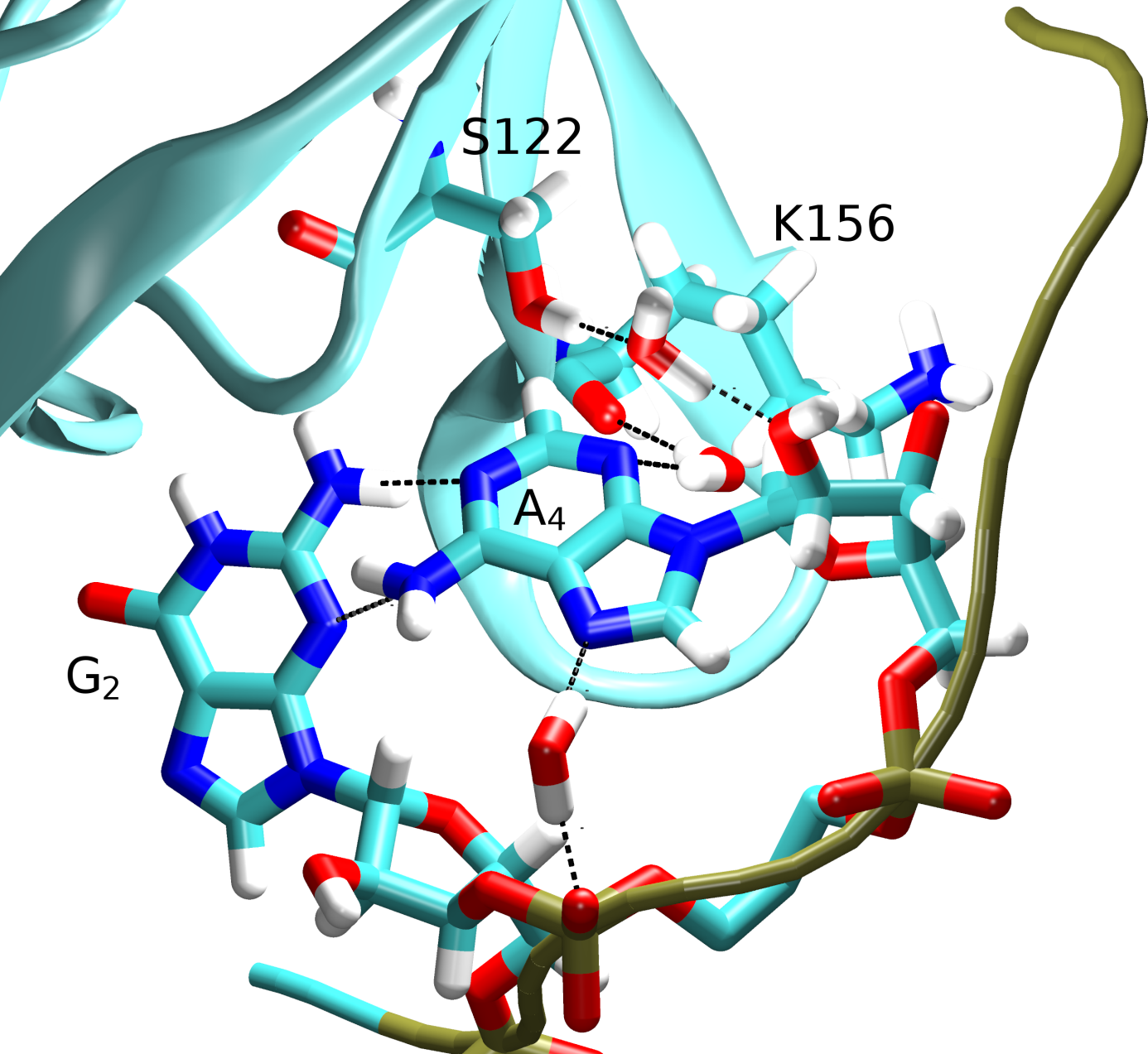


Figure S7. The A_4_ nucleotide does not form any direct H-bond interactions with the Fox-1 protein. However, the MD simulations showed it is engaged in three long-residency water bridges, two of which facilitate a contact with the protein. The H-bond interactions of the water bridges and the A_4_/G_2_ intramolecular base pair are indicated by black dotted lines. The interacting residues are labeled.


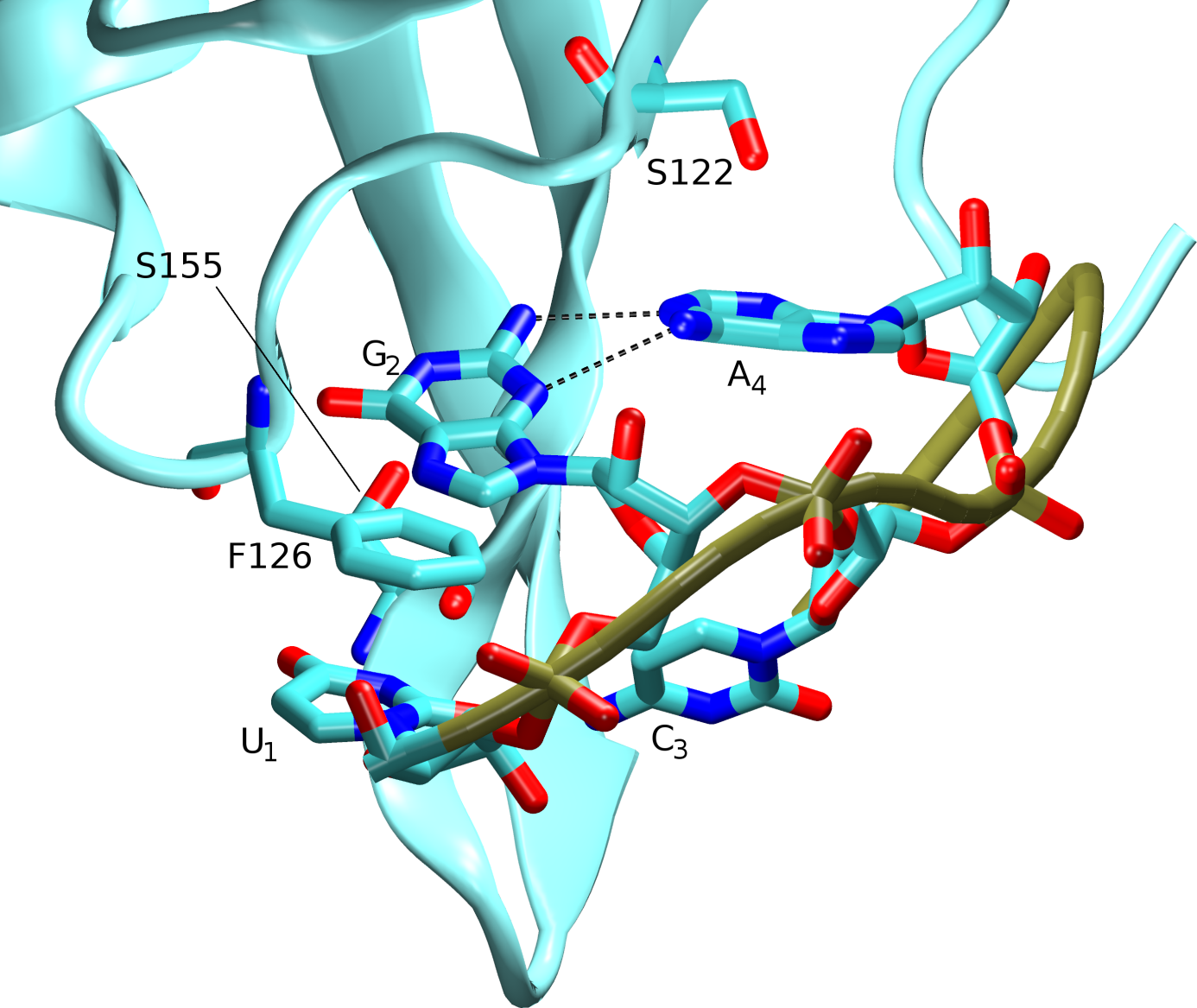


Figure S8. The hydrophobic pocket formed by the U_1_/F126/G_2_ stacking interactions and the C_3_/F126 van der Waals overlap. The A_4_/G_2_ intramolecular base pair is indicated by black dotted lines. The S155 and S122 residues are shown. The H-bond interactions of the A_4_/G_2_ intramolecular base pair are indicated by black dotted lines. For clarity, the hydrogen atoms are not shown.


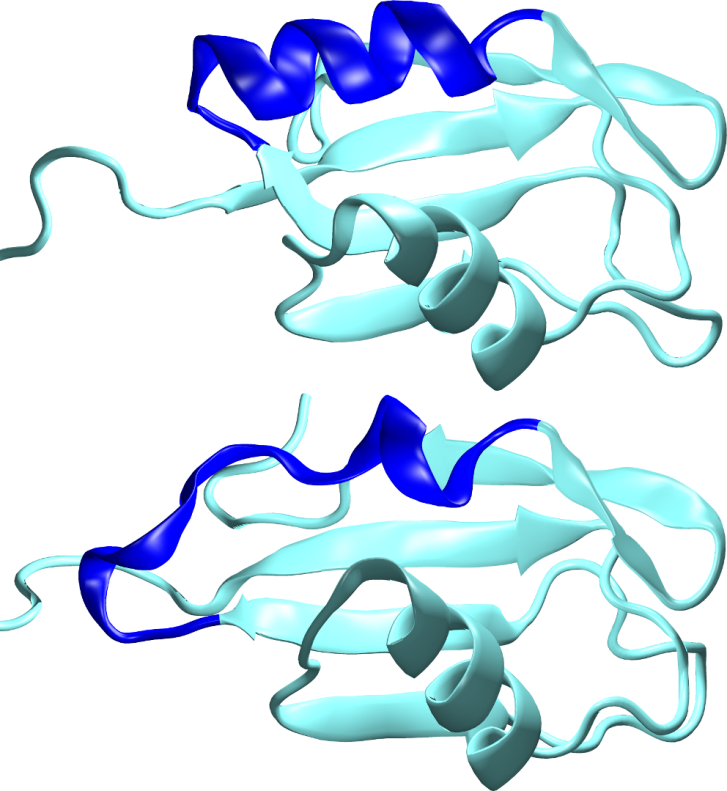


Figure S9. The experimental secondary structure of helix α2 (blue) of the Fox-1 RRM (top) was often unstable in the ff99SB and ff14SB simulations. The largest instability was observed at the end of the Fox-1(free*)_99_1 simulation (bottom).


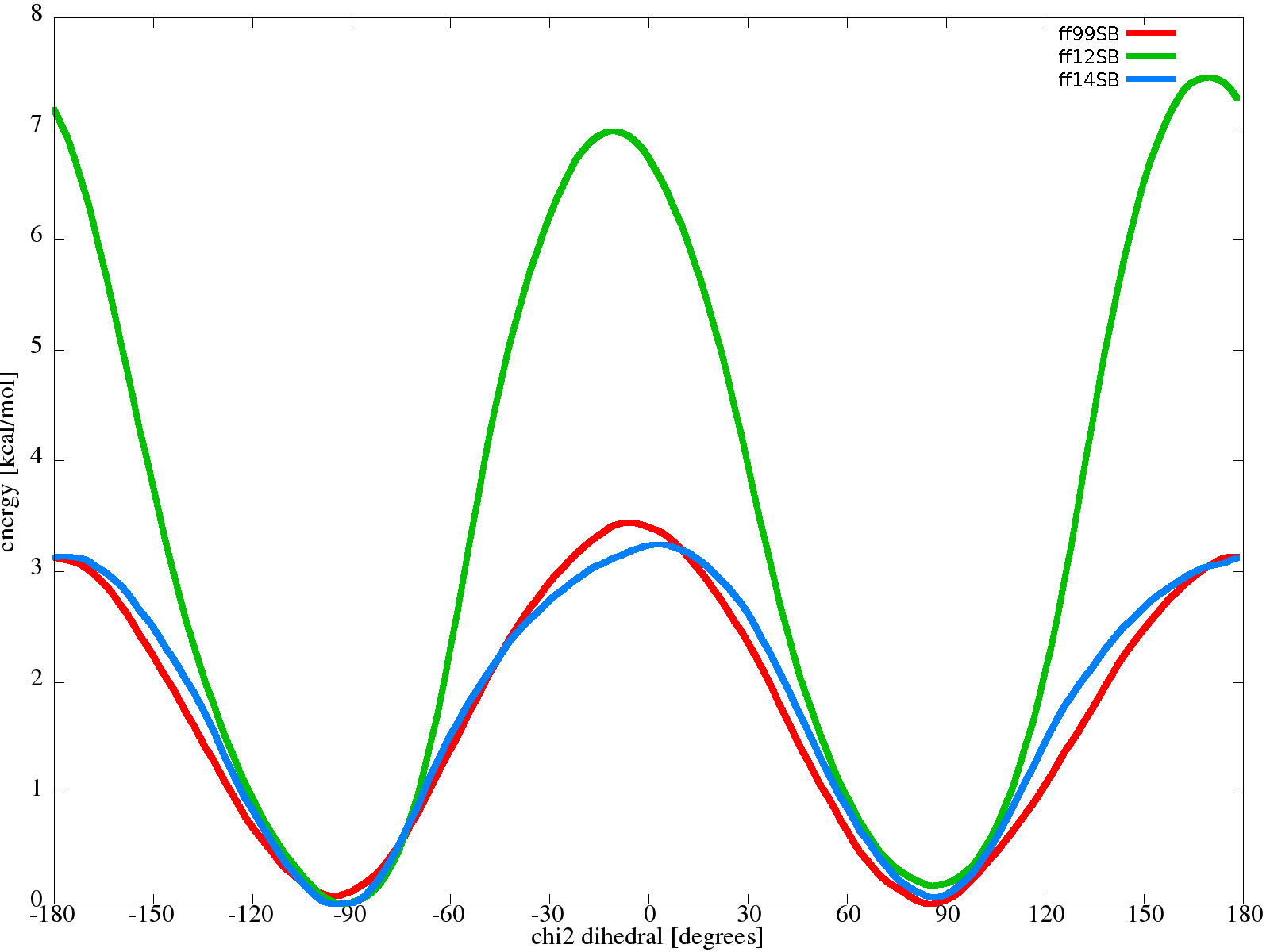


Figure S10. The dihedral profile of the χ_2_ side-chain dihedral (defined by atoms CA-CB-CG-CD1; describes rotation of the aromatic ring) of the phenylalanine. The energy profile was computed by rotating the phenylalanine χ_2_ dihedral in vacuum using the ABF (adaptive biasing force) method. With ff12SB (green) the energy barrier for the ring rotation is more than twice higher than with the ff99SB (red) or ff14SB (blue). In simulations of Fox-1 RRM, this results into more stable trajectories when using the ff12SB.
